# An Overlooked Prebiotic: Beneficial Effect of Dietary Nucleotide Supplementation on Gut Microbiota and Metabolites in Senescence-Accelerated Mouse Prone-8 Mice

**DOI:** 10.3389/fnut.2022.820799

**Published:** 2022-03-24

**Authors:** Ting Ding, Meihong Xu, Yong Li

**Affiliations:** Department of Nutrition and Food Hygiene, School of Public Health, Peking University, Beijing, China

**Keywords:** gut microbiota, metabonomic, SAMP8 mice, prebiotic, dietary nucleotide

## Abstract

Nucleotides (NTs) are regulatory factors in many biological processes and play important roles in the growth, development, and metabolism of living organisms. We used senescence-accelerated mouse prone-8 (SAMP8) to investigate the effects of NTs on the gut microbiota and metabolites. And the promoting effect of NTs on the growth of a probiotic (*Lactobacillus casei*) was explored through *in vitro* experiments. The results showed that the sequencing depth of 16S rDNA covered all microbial species in the feces of SAMP8. Supplementation with exogenous NTs to the diet enhanced the diversity of the gut microbiota, reduced the abundance of bacteria with negative effects on the body (such as *Verrucomicrobia, Ruminococcaceae, Akkermansia* and *Helicobacter*), and increased the abundance of the microbiota, which had beneficial effects on the mice (such as *Lactobacillus, Candidatus saccharimonas* and *Lachnospiraceae*_NK4A136_group). Metabonomic analysis showed that NT deficiency in the diet significantly affected metabolites in the mouse feces. The metabolites in mice supplemented with NTs tended to be normal (SAMR1). The differentially expressed metabolites caused by NT addition are involved in various pathways in the body, including linoleic acid metabolism, vitamin B6 metabolism, and histidine metabolism. Correlation analysis revealed a significant correlation between the gut microbiota and differentially expressed metabolites caused by the addition of NTs. *In vitro* experiments showed that NTs significantly promoted the growth, secretion of biofilm and extracellular polymeric substance of *L. casei*. NTs also promoted the ability of the crude extract of *L. casei* to resist the secretion of *Shigella* biofilm. Thus, NTs can regulate the abundance of the gut microbiota and alter the metabolic expression of the intestinal microbiome.

## Introduction

Aging is the process by which humans die following changes in the organizational structure, decreased functions, and reduced adaptability and resistance in cells over time. Aging, as a complex phenomenon, is a spontaneous and inevitable process. The incidence of diseases related to aging is significantly increasing, inflicting a heavy economic burden on individuals, families, and society. Anti-aging, health, and longevity have become the goals of many people, particularly women. This is also a high-priority topic in the research of life sciences in the 21st century. Although aging is inevitable, adopting good health care measures and using anti-aging products can effectively delay aging, improve the quality of life, and enable humans to achieve maximum longevity under existing conditions.

Studies have shown that aging results from various factors such as genes, free radicals, immune dysfunction, and gut microbiota imbalance (1–3). The gut microbiota is closely related to the health, disease, and aging status of the host (4, 5). The intestine, known as the “second genome,” is an extremely complex ecosystem. Many studies have shown that there is a close relationship between the gut microbiota and health (6). As age increases, the body's intestinal physiological function and diet structure change, body's immune function declines, and gut microbiota also changes accordingly, resulting in reduced microbial diversity that affects the aging state of the body, thus leading to a series of chronic diseases. The imbalance of gut microbiota may also lead to a series of symptoms, such as diarrhea, constipation, gastroenteritis, etc. In addition, metabolites of the gut microbiota may cause age-related diseases (7, 8). Therefore, it is of great significance to select appropriate substances to regulate the imbalance of gut microbiota caused by aging.

Nucleotides (NTs) are low-molecular weight and important components of cells and precursors of DNA and RNA. They play an important role in cell metabolism, protein synthesis, cell division, and body functions. For example, ATP transfers chemical energy to physiological processes, playing important roles in cell signaling transduction, such as that involving cAMP and cGMP, and are part of important cofactors for enzymatic reactions, such as NAD, coenzyme A, and NADP^+^. In addition, NTs and nucleosides are involved in coenzymes, activating substrates, and regulating and proliferating cells (9–12). Although the body can absorb and utilize NTs from the diet, NTs are “essential nutrients” under certain conditions, such as disease, intestinal injury, immune challenge, starvation, aging, rapid growth and development, limited nutrient intake, and endogenous synthesis and expression (13–21). These processes are involved in specific stages of life, including early life, before and after the reproductive years, and in later years. Studies have shown that NTs have many physiological functions such as immune regulation (22), resistance to infection (23), promotion of growth and development (24), maintenance of liver function (25), promotion of cell proliferation and differentiation (26), and improvement of memory function (27). Additionally, NTs show many other physiological activities, such as anti-fatigue and accelerating carbon turnover in the fundic stomach (28, 29). More importantly, studies have shown that NTs had the ability to main the intestinal function (30). NTs are likely to have the ability to regulate the imbalance of gut microbiota caused by aging. Therefore, *in vivo* experiments were conducted to explore the regulatory effect of NTs supplementation on gut microbiota and metabolites in SAMP8 mice, combined with16S rDNA with metabonomics analyses. Additionally, *in vitro* experiments were conducted to explore the promoting effect of NTs on the growth of a probiotic (*Lactobacillus casei*) and the antagonism of *L. casei* against intestinal pathogens. This study provides a foundation for applying NTs to regulate gut microbiota.

## Materials and Methods

### Animals and Treatment

A total of 200 SPF male senescence-accelerated mouse prone-8 (SAMP8) mice and senescence-resistant-1 (SAMR1) mice (as the model control) (weighing 18–22 g) were provided by Beijing Vitonglihua Experimental Animal Technology Co., Ltd. (Beijing, China). All mice had free access to food (American Institute of Nutrition Rodent Diets-93M (AIN-93M diet) and water. Dietary NTs (5′-NTs: 5′AMP: 5′CMP: 5′GMPNa_2_: 5′UMPNa_2_ = 16:41:19:24) were provided by Dalian Zhen-Ao Biotechnology Co., Ltd. (Dalian, China). The environment was maintained at 25 ± 1°C with a relative humidity of 50–60% and 12-h: 12-h light-dark cycle. Mice were allowed to adapt to the new environment for 7 days.

The SAMP8 mice were randomly divided into three groups with 50 mice in each group: NT-free group (group A), basal diet group (group B), and NT intervention group (group C) (1,200 mg/kg×bw), and 50 SAMR1 mice served as the model control group (Group D). One mouse in each cage. The mice in NT-free group were fed with the purified formula of AIN93M, and the basal diet was used for the controls (SAMP8 and SAMR1). For the NTs-treated group, NTs were added to the basal diet as an intervention. The ingredients of purified diet and basal diet can refer to Supplementary Tables 1, 2 as well as the differences of NTs content among the groups were shown in the tables. The NTs levels in NT-free, basal and NTs-supplied diets were shown in Supplementary Table 3. Age-related changes in the body and behavior of mice were measured by aging degree scores according to the method of Hosokawa et al. (31). In month 10, when aging was observed in the mice, their feces were collected aseptically. The aseptic cushion was replaced on the day of sampling to reduce contamination of the samples by external microorganisms. The feces of six mice from each group were randomly sampled and ensured that the feeding environment, NTs intervention and sampling time were as consistent as possible to eliminate the effects cage to cage variations. The samples were transported to Shanghai Baiqu Biomedical Technology Co., Ltd. (Shanghai, China) with dry ice for 16S rDNA and metabonomic detection.

### Detection of 16S rDNA in Mouse Feces

Total bacterial DNA was extracted using a TGuide S96 Magnetic Soil/Stool DNA Kit (Tiangen Biotech (Beijing) Co., Ltd, China) according to the manufacturer's instructions. DNA quality and quantity were assessed based on the 260/280 nm and 260/230 nm ratios. Thereafter, DNA was stored at −80°C for further analysis. The V3-V4 region of the bacterial 16S rRNA gene was amplified using a common primer pair (forward primer, 5′-ACTCCTACGGGAGGCAGCA-3′; reverse primer, 5′- GGACTACHVGGGTWTCTAAT-3′) combined with adapter sequences and barcode sequences. PCR amplification was performed in a total volume of 50 μL containing 10 μL of bμffer, 0.2 μL of Q5 High-Fidelity DNA Polymerase, 10 μL of High GC Enhancer, 1 μL of dNTP, 10 μM of each primer, and 60 ng of genome DNA. The thermal cycling conditions were as follows: initial denaturation at 95°C for 5 min, followed by 15 cycles at 95 °C for 1 min, 50°C for 1 min, and 72°C for 1 min, with final extension at 72°C for 7 min. The PCR products from the first step of PCR were purified using VAHTSTM DNA Clean Beads (Vazyme Biotech, Nanjing, China). A second round of PCR was performed in a 40-μL reaction volume containing 20 μL 2 × Phμsion HF MM, 8 μl ddH_2_O, 10 μM each primer, and 10 μL PCR products from the first step. Thermal cycling conditions were as follows: initial denaturation at 98°C for 30 s, followed by 10 cycles at 98°C for 10 s, 65°C for 30 s, and 72°C for 30 s, with final extension at 72°C for 5 min. Finally, all PCR products were quantified using Quant-iT™ dsDNA HS Reagent and pooled. High-throughput sequencing analysis of bacterial rRNA genes was performed on the purified, pooled sample using an Illumina Hiseq 2500 platform (2 × 250 paired ends; San Diego, CA, USA). After sequencing, the data were preprocessed, including raw read filtering, high-quality reads splicing and block removal, and the final effective data (effective reads) were obtained. For information analysis, we divided the features and performed diversity analysis, difference analysis, correlation analysis, and function prediction analysis. Among them, operational taxonomic unit analysis clustered the sequences at a 97% similarity level, and filtered operational taxonomic units with 0.005% of the total sequence number as a threshold. The 16s: Silva (release128, http://www.arb-silva.de) database was selected, and species annotation was performed using RDP classifier (confidence threshold = 0.8). The dilution curve was drawn, and the alpha-diversity and β-diversity of the samples were calculated to evaluate bacterial diversity. Alpha diversity was expressed using the Chao1 richness estimator, PD_whole_tree index, Ace richness estimator, Shannon–Wiener diversity index, and Simpson diversity index. β-Diversity was represented using principal coordinate analysis (PCoA) and non-metric multidimensional scaling (NMDS). Linear discriminant analysis (LDA) was used to analyze species showing significant differences in abundance among groups.

### Metabonomics Analysis of Mouse Feces

The extraction steps of metabolites from mouse feces were as follows: 25 mg of sample was weighed, and 500 μL of extraction solution (methanol: acetonitrile: water = 2: 2: 1 (V/V), including isotope-labeled internal standard mixture) were added. The sample was then ground for 4 min at 35 Hz and treated with ultrasound for 5 min in an ice water bath. The extraction procedure was repeated three times, and the sample was stored at −40°C for 1 h. The sample was centrifuged at 12,000 rpm for 15 min at 4°C. The supernatant was placed into the injection bottle for detection, and all samples were mixed with the same amount of supernatant to form quality control (QC) samples and tested.

The target compounds were separated using ultra-performance liquid chromatography (Vanquish, Thermo Fisher Scientific, Waltham, MA, USA) (liquid chromatographic column: Waters ACQUITY UPLC BEH Amide, 2.1 × 100 mm, 1.7 μm, Milford, MA, USA). Phase A used for HPLC was ultrapure water containing 25 mmol/L ammonium acetate and 25 mmol/L ammonia water. Phase B consisted of acetonitrile. The sample disk temperature was: 4°C, and the injection volume was 3 μL. A Thermo Q exictive HFX mass spectrometer (Thermo Fisher Scientific) was used to collect primary and secondary mass spectrum data under control of an xcalibur (Thermo Fisher Scientific). The parameters were as follows: sheath gas flow rate: 30 Arb; Aux gas flow rate: 25 Arb; capillary temperature: 350°C; full ms resolution: 60,000; tandem mass spectrometry resolution: 7,500; collision energy: 10/30/60 in normalized collision energy mode; spray voltage: 3.6 kV (positive) or −3.2 kV (negative).

After peak recognition, peak extraction, peak alignment, and integration, the original data were matched with the secondary mass spectrometry database built by BIOTREE dB (v2.1) for substance annotation. The cutoff value of the algorithm was set to 0.3.

### Correlation Analysis

After obtaining the content information of differential flora identified from the 16S rDNA and that of differentially expressed metabolites obtained using metabonomics detection, the “Spearman” algorithm was used to perform correlation analysis. To further verify the authenticity of the correlation obtained using correlation coefficient analysis, the flora and metabolites were analyzed using a scatter plot to remove the strong correlation effect of false-positives.

### *In vitro* Experiment

*Lactobacillus casei* Q1 was stored in the refrigerator at −80 °C in our laboratory. A total of 1% bacteria (v/v) were inoculated in MRS broth medium with shaking (160 rpm) at 37°C for 24 h. MRS medium was served as the blank control. Then, 1% of *L. casei* and 1%, 2%, 3% and 4% (g/100 mL) of NTs were added to MRS medium, respectively and the bacteria was cultured at 37°C for 24 h, and the bacteria were counted by double dilution method. The growth of *L. casei* was non-linear fitted by modified Gompetz model and modified Logistic model.

Modified Gompertz model:


(1) $$\begin{array}{l} \text{log}(N_{t})=\text{log}(N_{0})+\text{log}\left(\frac{N_{\text{max}}}{N_{0}}\right)\\ \;\times \text{exp}\{-\text{exp}\left\lfloor \frac{\mu_{\text{max}}\times 2.718}{\text{log}(N_{\text{max}}/N_{0})}\times (\lambda -t)+1\right\rfloor \} \end{array}$$

*t* is the time (h), N_t_ is the number of bacteria at time *t*, N_max_ and N_0_ are the maximum and initial number of bacteria (CFU/mL). μ_max_ is the maximum specific growth rate (h^−1^), λ is the growth delay time (h).

Modified Logistic model:


(2) $$\begin{array}{l} \text{log}\left(N_{t}\right)=\text{log}\left(N_{0}\right)+\frac{A}{1+\text{exp}\left(4\;*\mu_{max}\frac{\lambda -t}{A}+2\right)}. \end{array}$$

N_t_ is the number of bacteria at time *t* (CFU/mL); N_0_: initial colony count (CFU/mL); μ_max_: maximum specific growth rate (h^−1^); A: Fitting parameters; λ is the growth delay time (h).

The effects of NTs on *L. casei* biofilm, extracellular polymeric substance and crude extract of *L. casei* against *Shigella* biofilm were studied according to the previous literature (32). In addition, *L. casei* and *Salmonella enterica* strains were cultured to the same concentration with shaking (160 rpm) at 37°C for 24 h. *L. casei* was centrifuged at 4°C (10,000 r/min) for 10 min (SIGMA3K15 Desktop high-speed low-temperature freezing centrifuge, Sigma, Germany). The supernatant was discarded, and the cells were washed with sterile water for 3 times, and then re-suspended with the same amount of sterile water. After *S. enterica* and fresh LB broth medium were mixed in the ratio of 1:100 (v/v), 0.5 mL of *L. casei* suspension was added to make the co culture system of the two strains. 4.0% NTs mixture (1:1:1:1) was added to the co culture system, and the medium without NTs was served as control. The bacteria was cultured at 28°C for 24 h. *L. casei* and *S. enterica* under the same culture conditions were used as blank control. The target gene (Supplementary Table 6) was determined by real-time fluorescence quantitative PCR. Reaction system 20 μL: 2 × SYBR Green PCR Master Mix 10 μL; ultrapure water 4 μL; the upstream and downstream primers were 0.5 μL, respectively; cDNA 5 μL. The PCR reaction conditions were as follows: pre denaturation at 95°C for 3 min; denaturation at 95°C for 10 s; annealing at 55°C for 20 s; extend at 72°C for 20 s; the melting curve was established in the range of 65–95°C after staying at 75°C for 5 s and 40 cycles.

### Statistical Analysis

Data were processed using a statistical software package. The measurement data were expressed as the mean ± standard deviation, analyzed by one-way analysis of variance with SPSS Statistics 16.0 software (SPSS, Inc., Chicago, IL, USA). Differences were considered as significant at *P* < 0.05.

## Results

### 16S rDNA Results

To explore the effect of dietary NTs on the gut microbiota of SAMP8 mice, the structure of the microflora in the mouse feces was analyzed. The results showed that 1,868,237 pairs of reads were obtained from 24 samples. After QC and splicing, 1,847,859 clean reads were obtained. At least 57,590 clean reads were obtained from each sample, and the operational taxonomic unit range of the samples was 276–392.

**Figure 1:**
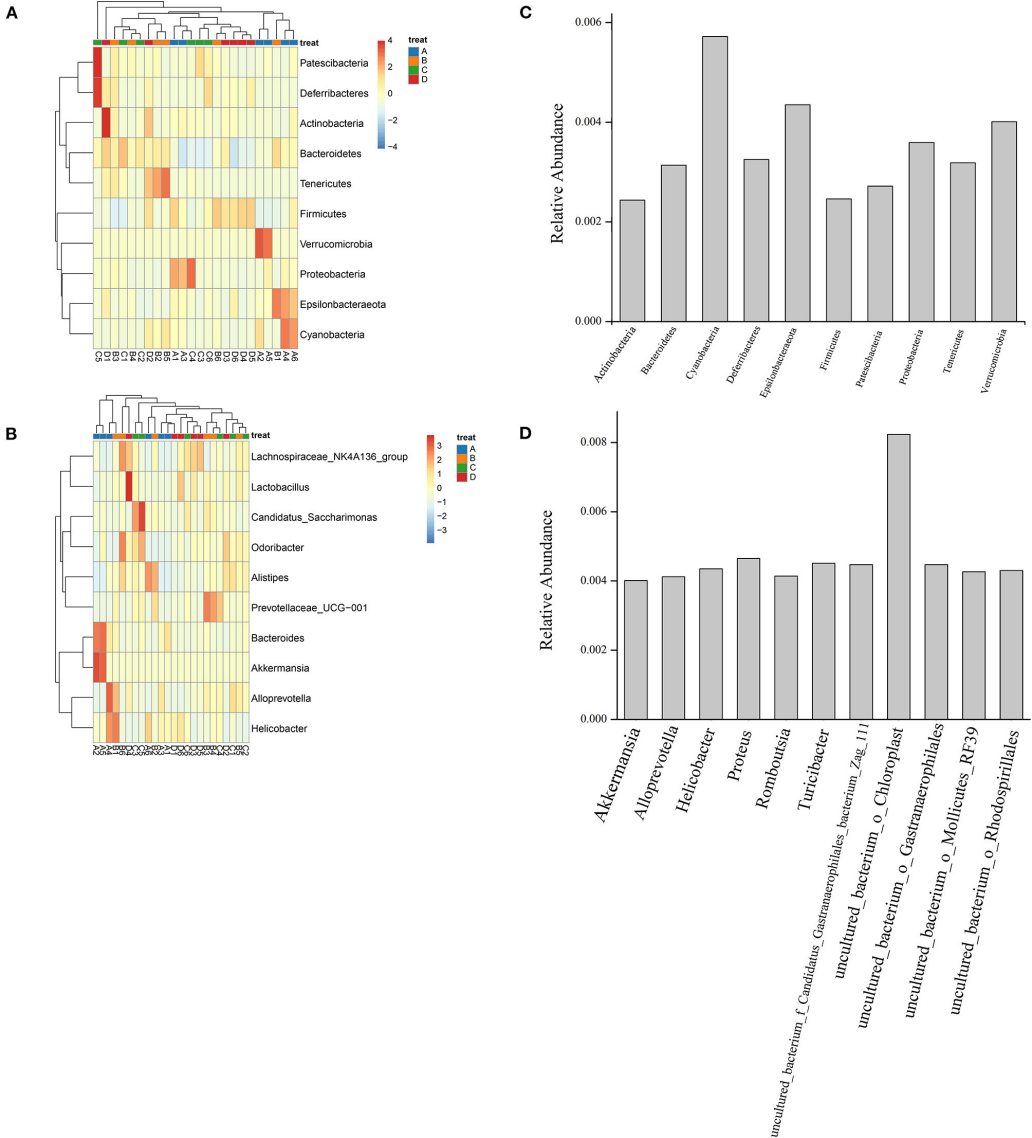
Species distribution heatmap at phylum **(A)** and genus level **(B)** and relative abundance of aging related bacteria at phylum **(C)** and genus level **(D)** of gut microbiota of mouse fecal in four groups. A1-A6: NT-free group; B1-B6: basal diet group; C1-C6: NT intervention group; D1-D6: model control group.

A heatmap of the species distribution at the phylum and genus levels is shown in Figures 1A,B. As shown in the figure, Bacteroidetes, Patescibacteria, Cyanobacteria, Actinobacteria, Firmicutes, Verrucomicrobia, Deferribacteres, Tenericutes, Epsilonbacteraeota, and Proteobacteria were the most abundant bacteria in the four groups. Bacteroides and Firmicutes showed high species abundance in the four groups. Bacteroidetes and Firmicutes accounted for a large proportion of the four groups. At the genus level, the bacteria with high relative abundance were *Lachnospiraceae*_NK4A136_group, *Odoribacter, Alistipes, Prevotellaceae*_UCG-001, *Bacteroides, Alloprevotella, Helicobacter, Candidatus saccharimonas, Akkermansia*, and *Lactobacillus*. In group A (NT-free group), Bacteroides showed the highest relative abundance (13.36 ± 9.71%), followed by Akkermansia (13.00 ± 19.60%). Bacteria with the highest relative abundance were *Lachnospiraceae*_NK4A136_group (9.98 ±8.77%) in group B (basal diet group), followed by *Alistipes* (8.42 ± 4.28%). The *Lachnospiraceae*_NK4A136_group was the most abundant bacteria in group C (NT intervention group) (8.50 ± 4.63%), followed by *Candidatus_Saccharimonas* (8.35 ± 8.02%). In group D (model control group), *Lachnospiraceae*_NK4A136_group was the most abundant bacteria (12.51 ± 8.49%), followed by *Lactobacillus* (5.76 ± 5.08%). Relative abundance of aging related bacteria at phylum level and genus level was showed in Figures 1C,D. The abundance of Cyanobacteria, Epsilonbacteraeota and Verrucomicrobia associated with aging was high at the phylum level. At the genus level, the bacteria with high abundance associated with aging were uncultured_bacterium_o_*Chloroplast* and proteus.

**Figure 2:**
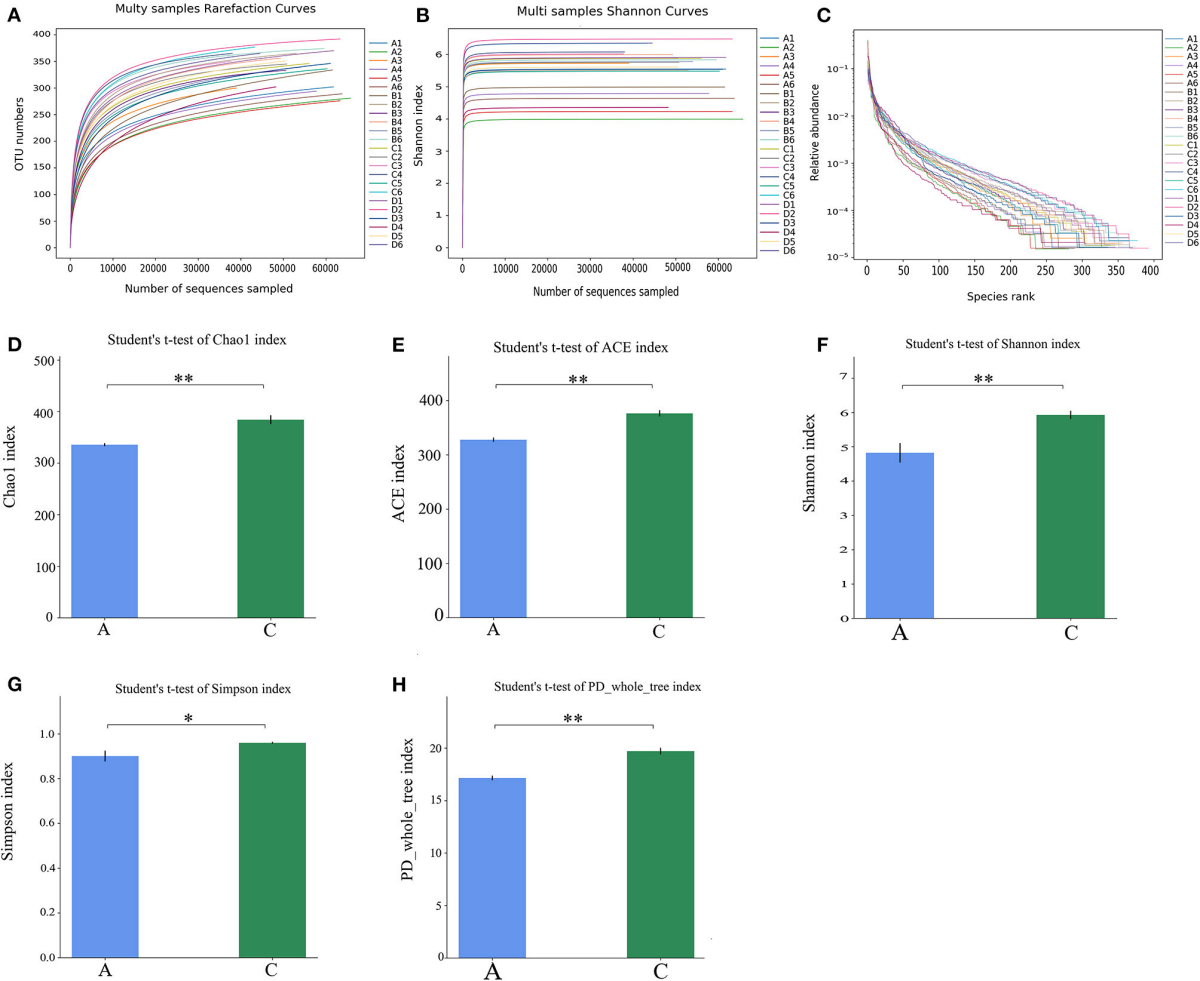
Mouse fecal sample dilution curve **(A)**, Shannon index **(B)**, Rank-Abundance curve **(C)** and histogram of difference between NT-free group and NT intervention group in Alpha diversity index **(D–H)**. **(A)**: NT-free group; **(B)**: basal diet group; **(C)**: NT intervention group; **(D)**: model control group. ^*^p < 0.05, ^**^p < 0.001.

The alpha diversity of each sample is shown in Figure 2. As shown in Figure 2A, the dilution curve tended to be flat, indicating that the 16S rDNA sequencing depth covered all species in the sample. In Figure 2B, the curves tended to be flat, indicating that most microbial species information was covered in the samples. In Figure 2C, the length of the NT-free group was smaller than that of the other groups on the horizontal axis, indicating that the species composition of the NT-free group was not as rich as that in the other three groups, and the degree of uniformity was the lowest. These results indicate that NTs significantly affected the richness and evenness of species in the mouse feces. In Figures 2D–H, the abscissa is the group name and ordinate is the alpha diversity index value. The ACE index, Chao-1 index, Shannon index and PD_whole_tree index of the NT-free group were extremely significant different from that of NT intervention group, indicating that NTs significantly affected the fecal microbial abundance. The Simpson index showed a significant difference between the NT-free and NT intervention groups. These results indicated that the presence or absence of NTs in the diet plays an important role in fecal species diversity. Higher Shannon and Simpson indices indicated higher species diversity. The results showed that the fecal species diversity of mice in the NT group was higher than that in the NT-free group. Therefore, dietary NTs can increase the diversity of the gut microbiota in mice.

**Figure 3:**
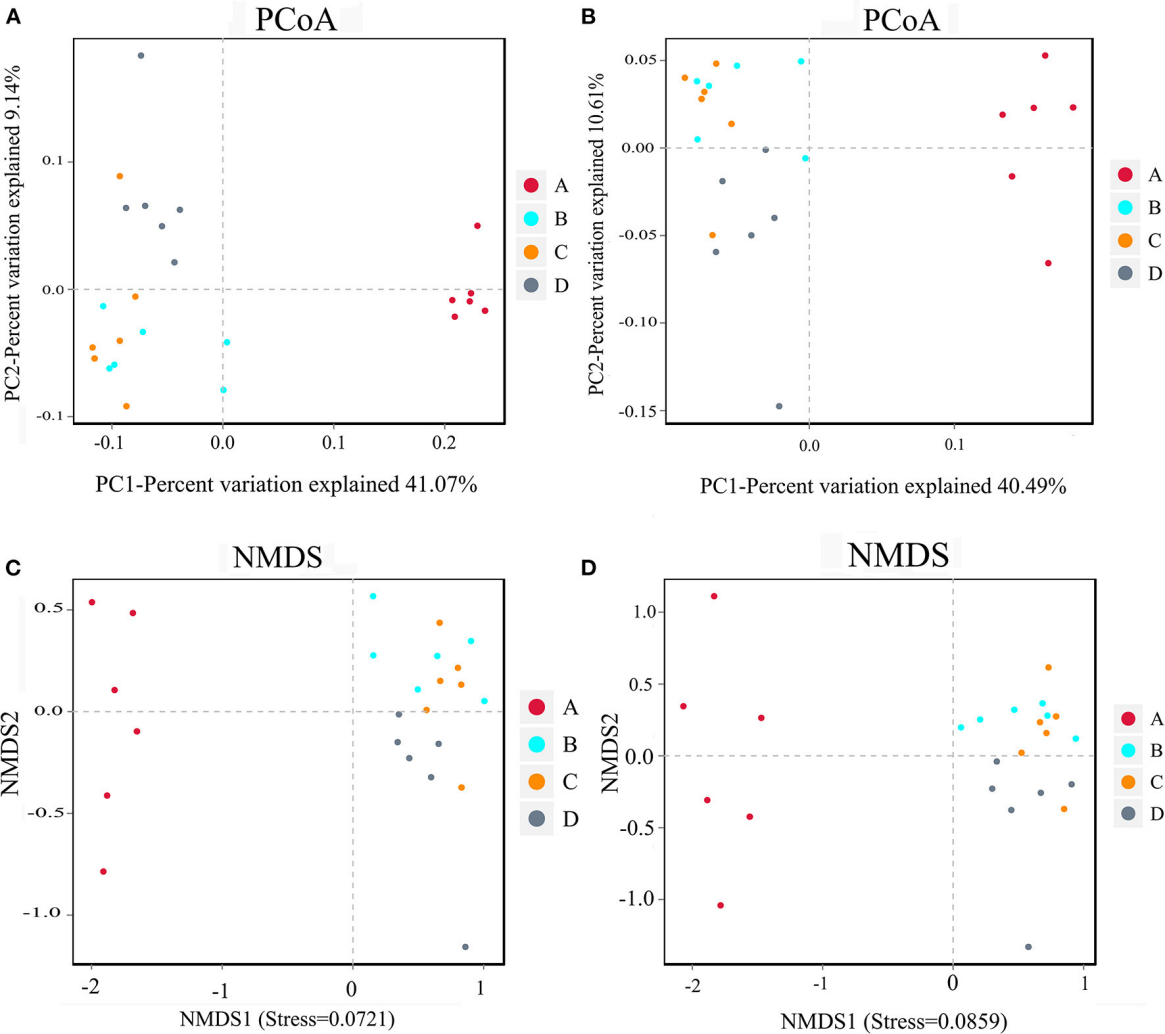
PCoA analysis chart and NMDS analysis chart of the bacteria in mouse feces. **(A,C)** Binary jaccard; **(B,D)** unweighted unifrac. A: NT-free group; B: basal diet group; C: NT intervention group; D: model control group.

The results of beta diversity analysis of the samples were mainly shown in PCoA and NMDS diagrams (Figure 3). The PCoA diagram shows that the closer sample points indicated greater similarity. All data points in the nucleoside-free group were clustered, indicating that the similarity between samples was high. The data points in the NT-free group were on the right side of the data score graph; however, those of the other three groups were on the left side of the graph. The NMDS diagram showed the opposite trend. These results indicate that there were significant differences between the NT-free group and other three groups in the gut microbiota of mouse feces. PCoA and NMDS were used to distinguish samples in each group, and exogenous NTs significantly affected the gut microbiota of mice.

**Figure 4:**
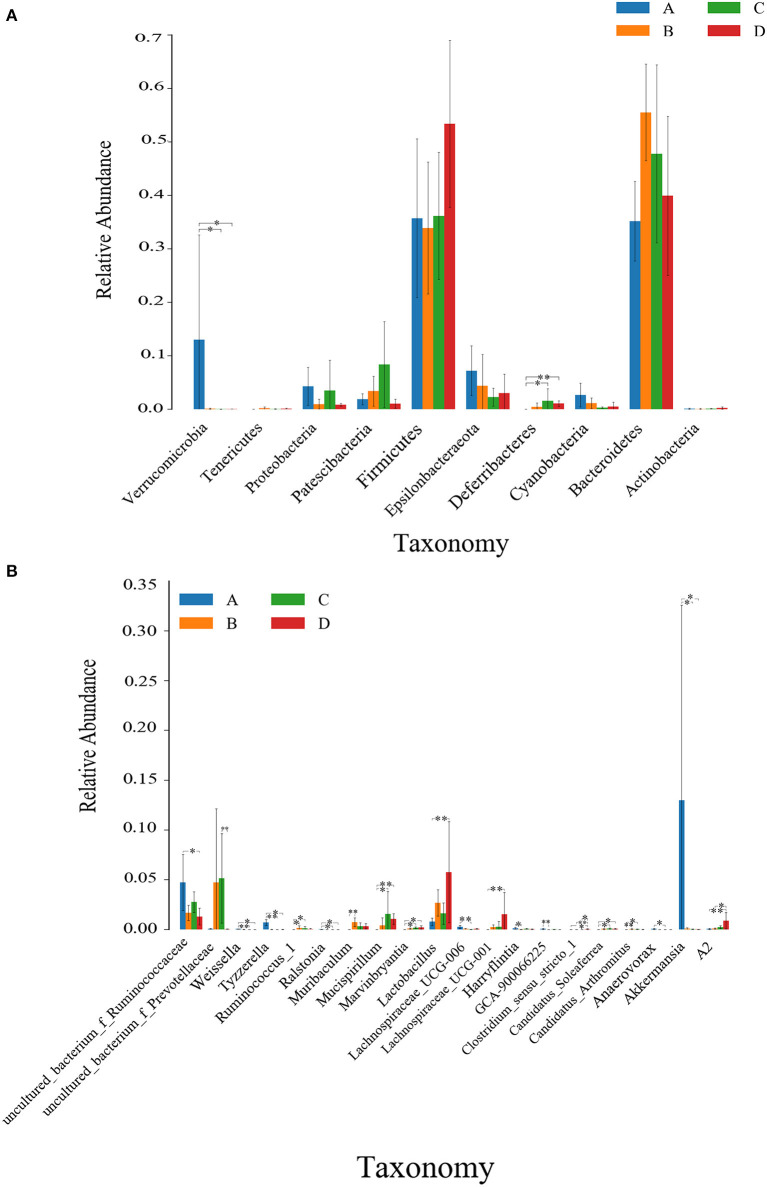
Histogram of Kruskal Wallis rank sum test at phylum **(A)** and genus levels **(B)** between groups. ^*^ on the column indicating significant difference (*P* < 0.05); and ^* *^ indicating extremely significant difference (*P* < 0.01). A: NT-free group; B: basal diet group; C: NT intervention group; D: model control group.

Kruskal Wallis rank sum test was used to analyze the significance of differences between groups. As shown in Figure 4, at the phylum level, the relative abundance of Verrucomicrobia was highest in the NT-free group, showing a significant difference from the NT intervention group and model control group (*P* < 0.05). The relative abundance of Deferribacteres was lowest in NT-free group, showing a significant difference from the NT intervention group and an extremely significant difference compared to the model control group (*P* < 0.01). At the genus level, the relative abundance of *Lactobacillus* was lowest in the NT-free group, which was significantly different from that in model control group (*P* < 0.01). The relative abundance of uncultured_bacterium_f_*Ruminococcaceae* was highest in the NT-free group, showing a significant difference from the model control group (*P* < 0.05). The relative abundance of *Akkermansia* in the NT-free group was highest, showing a significant difference from the NT intervention group and model control group (*P* < 0.05).

**Figure 5:**
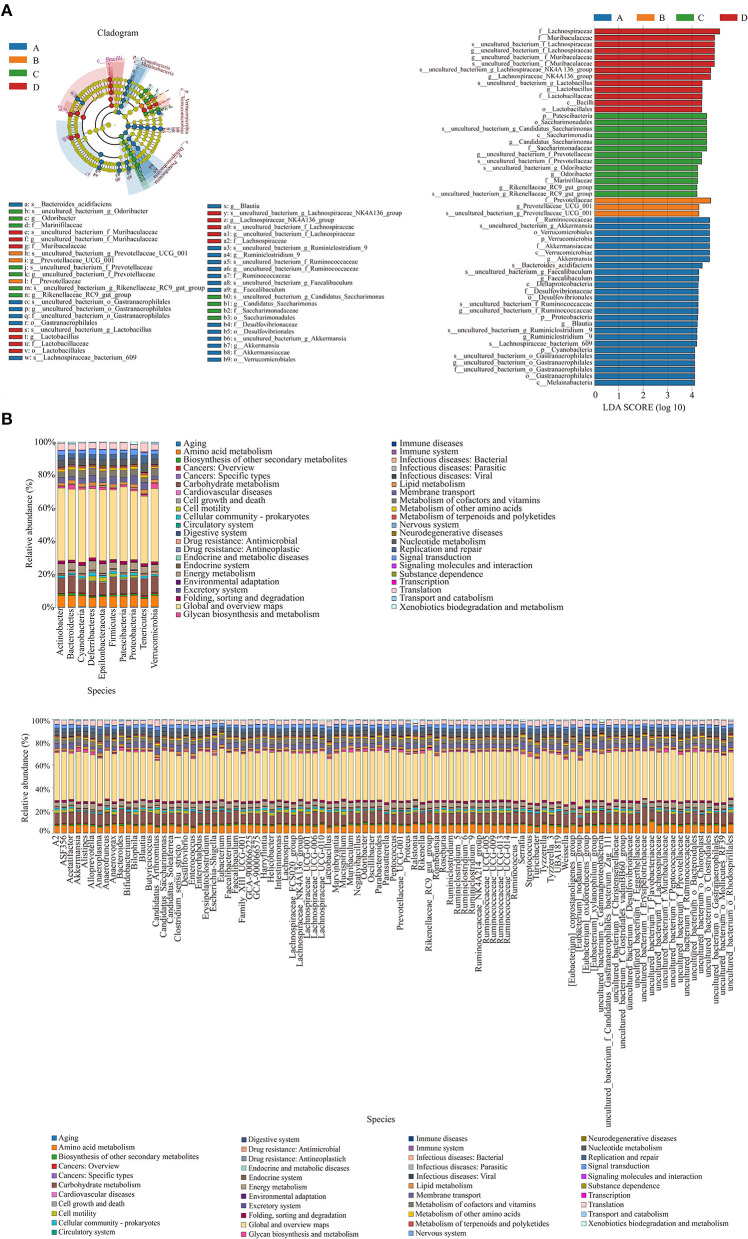
Evolutionary branching diagram of LEfSe analysis and histogram of LDA value distribution of gut microbiota **(A)** and KEGG metabolic pathway histogram at phylum level and genus level **(B)** of functional genes of gut microbiota in SAMP8 and SAMR1 mice. A: NT-free group; B: basal diet group; C: NT intervention group; D: model control group. In the evolutionary branching diagram, the circle radiated from inside to outside represented the taxonomic level from phylum to species. The diameter of the small circle was proportional to the relative abundance. The species with no significant difference was represented by yellow, and the other species with the highest abundance were colored according to the group with the highest abundance.

Figure 5A shows the species whose LDA score was more significant than the set value (4.0). In the NT-free group, f_Ruminococcaccac, s_uncultured_bacterium_g_Akkermansia, o_Verrucomicrobiales, and p_Verrucomicrobia were the most influential bacteria; f_Prevotellaceae and g_Prevotellaceae_UCG_001 were influential bacteria in the basal diet group; p_Patescibacteria, o_Ssccharimonadales, and s_uncultured_bacterium_g_Candidatus_Saccharimonas were influential bacteria in the NT intervention group, and f_Lachnospiraceae and f_Muribaculaceae were influential in the model control group. To identify species with significant differences in the feces of mice in each group, LDA effect size analysis was performed among groups. Bacteroides, Gastranaerophilales, Lachnospiraceae, Ruminococcaceae, Faecalibaculum, and Desulfovibrionaceae were enriched in the NT-free group, Prevotellaceae was mainly enriched in the basal diet group; Odoribacter, Marinifilaceae, Prevotellaceae, Rikenellaceae, and Saccharimonadia were mainly enriched in the NT intervention group, and Muribaculaceae and Lactobacillaceae were mainly enriched in the model control group.

The differences and changes in functional genes of the microbial community in different groups were evaluated using Kyoto Encyclopedia of Genes and Genomes metabolic pathway analysis. Figure 5B shows the prediction analysis of gene function, revealing that amino acid metabolism, carbohydrate metabolism, energy metabolism, membrane transport, metabolism of cofactors and vitamins, nucleoside metabolism, signal transduction, and translation were significantly affected by exogenous NTs. Therefore, exogenous NTs may affect the relative abundance of bacteria that are closely related to the above metabolic pathways to regulate the gut microbiota of mice.

**Figure 6:**
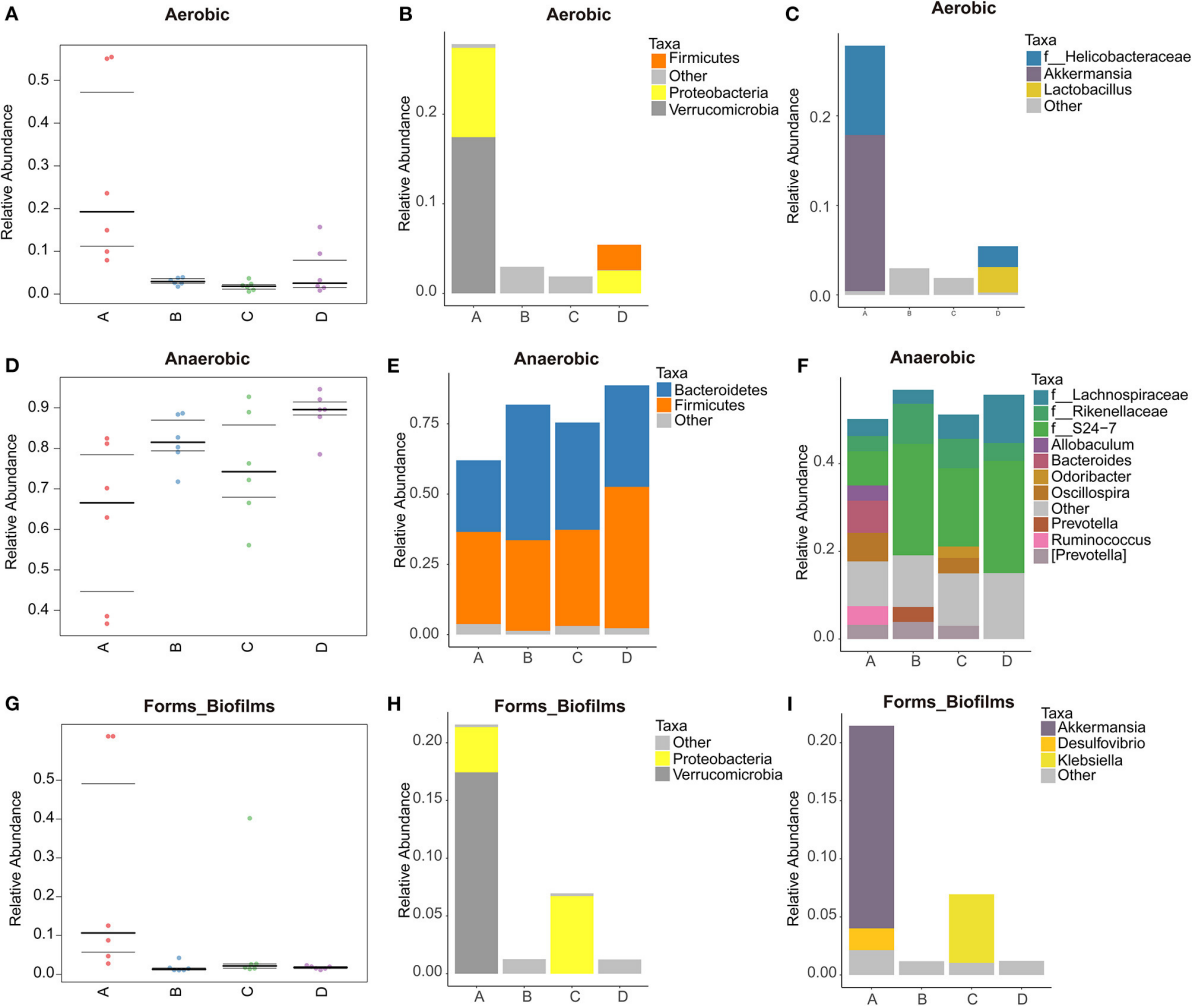
Phenotype prediction and species histogram of BugBase. **(A,D,G)**: phenotype prediction map; **(B,C,E,F,H,I)**: species histogram. A: NT-free group; B: basal diet group; C: NT intervention group; D: model control group.

BugBase phenotypic prediction analysis was shown in Figure 6. It can be seen from Figure 6A that the relative abundance of aerobic bacteria in NT-free group is large, mainly the bacteria of Verrucomicrobia and Proteobacteria (Figure 6B). At the genus level (Figure 6C), the aerobic bacteria in NT-free group contain high abundance of f_*Helicobacteraceae* and *Akkermansia*. *Helicobacteraceae* is an important microorganism causing gastrointestinal diseases. The results showed that NT deficiency would increase the abundance of pathogenic microorganisms such as *Helicobacteraceae*. It should be noted that model control group contains *Lactobacillus*, while basal diet group does not contain *Lactobacillus* in SAMP8 mice, indicating that aging will affect the gut microbiota of mice. In Figure 6D, the abundance of anaerobic bacteria in NT-free group is the lowest and that in model control group is the highest. And anaerobic bacteria are mainly *Firmicutes* and *Bacteroidetes*. As can be seen from Figure 6E, the abundance of *Bacteroidetes* in NT-free group is the lowest, indicating that NT deficiency significantly affects the abundance of *Bacteroidetes*. Figure 6G shows that NT-free group has the highest abundance of bacteria that can form biofilm. At the phylum level (Figure 6H), the biofilm formed in NT-free group was mainly Verrucomicrobia and Proteobacteria. At the genus level, biofilm formation in NT-free group is mainly concentrated in *Akkermansia* and *Desulfovibrio*, while NT intervention group was mainly *Klebsiella*. *Klebsiella* is mainly parasitic in the respiratory tract or intestinal tract of human and animals. It rarely endangers human and livestock and poultry under normal conditions. It can cause human and livestock and poultry diseases only under special circumstances. These results indicated that NT deficiency would indeed increase the abundance of pathogenic bacteria that can produce biofilm and easily cause gastrointestinal inflammation.

### Metabonomics Results

The original data obtained in positive ion mode included three QC samples and 24 experimental samples, and 17,480 peaks were extracted. A total of 13,459 peaks was retained after pretreatment. As shown in Supplementary Figure 1, the metabolite spectra of fecal samples from different groups of mice significantly differed.

**Figure 7:**
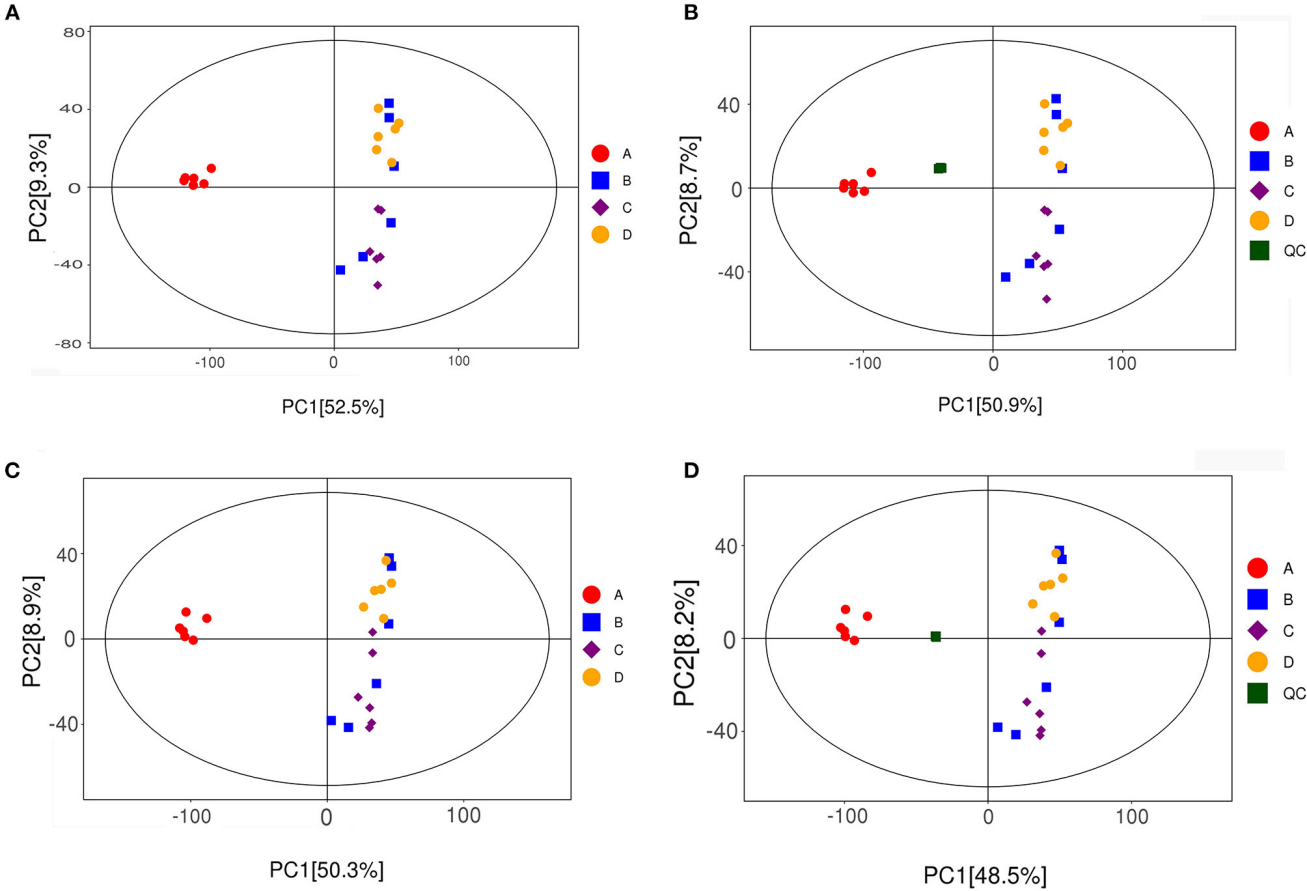
Scatter plot of PCA scores of all fecal samples (including QC samples). **(A,B)**: positive ion mode; **(C,D)**: negative ion mode. A: NT-free group; B: basal diet group; C: NT intervention group; D: model control group.

PCoA can reveal the internal structure of data to better explain the data variables. The PCoA score scatter plots of all samples (including QC samples) are shown in Figure 7. PCoA analysis of the fecal data of mice in each group showed that all samples were in the 95% Hotelling's t-squared ellipse. The NT-free group was significantly separated from the other groups, indicating that the metabolites in the feces of this group significantly differed from those of the other groups. The data points of SARM-1 mice (model control group) were distributed in the upper right corner of the graph. In contrast, those of SARMP-8 mice in the basal diet group were distributed in the upper right corner and lower right corner of the graph, indicating that aging affects the secretion of metabolites in mice. The data points for the NT intervention group were closer to those in the model control group, indicating that the metabolites of mice supplemented with NTs in the diet tended to be normal.

**Figure 8:**
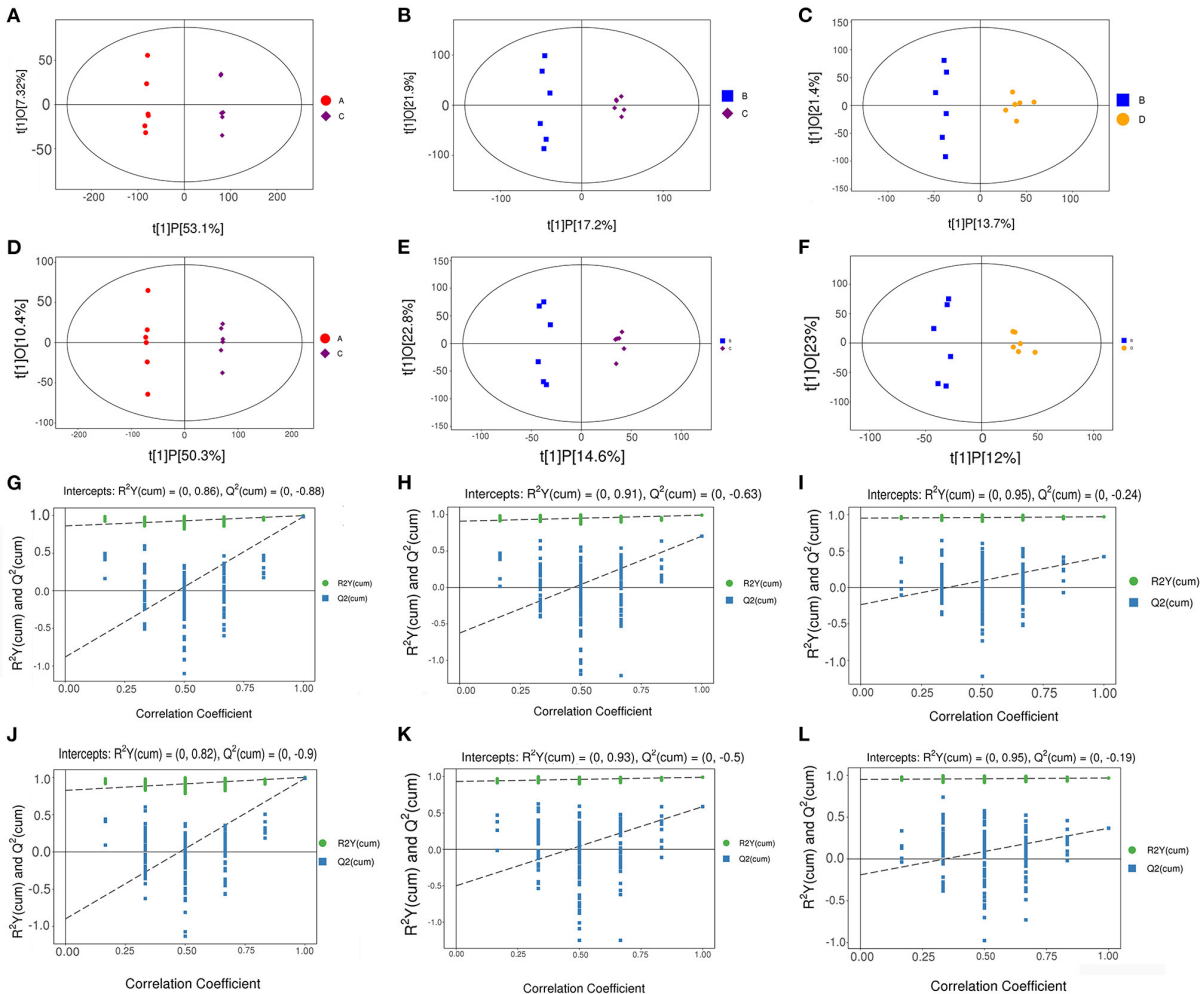
Scatter plot of OPLS-DA model **(A–F)** and permutation test of OPLS-DA model **(G–L)**. **(A–C), (G–I)**: Positive ion mode; **(D–F), (J–L)**: Negative ion mode. **(A,D,G,J)**: NT-free group compared with NT intervention group; **(B,E,H,K)**: basal diet group compared with NT intervention group; **(C,F,I,L)**: basal diet group compared with model control group.

Orthogonal projections to latent structures-discriminant analysis (OPLS-DA) were used to analyze the results; the scatter plot and permutation test results of the OPLS-DA model are shown in Figure 8. The OPLS-DA scatter plot shows that the two groups of samples were well-distinguished, and all the samples were within 95% Hotelling's *t*-squared ellipse. In the permutation test of the OPLS-DA model, the R^2^Y values of NT-free group compared with NT intervention group, basal diet group compared with NT intervention group, and basal diet group compared with the model control group were 0.999, 0.992, and 0.968, respectively. R^2^Y was very close to 1, indicating that the established model conformed to real sample data. Q^2^ indicates the reliability of the predicted results. Q^2^ was very close to 1, indicating that the original model had a good fitting degree and prediction ability, which may explain the differences between the two sample groups. In the permutation test, the intercept between the regression line and longitudinal axis of Q^2^ was less than zero, indicating that the original model was effective and that there was no overfitting phenomenon.

**Figure 9:**
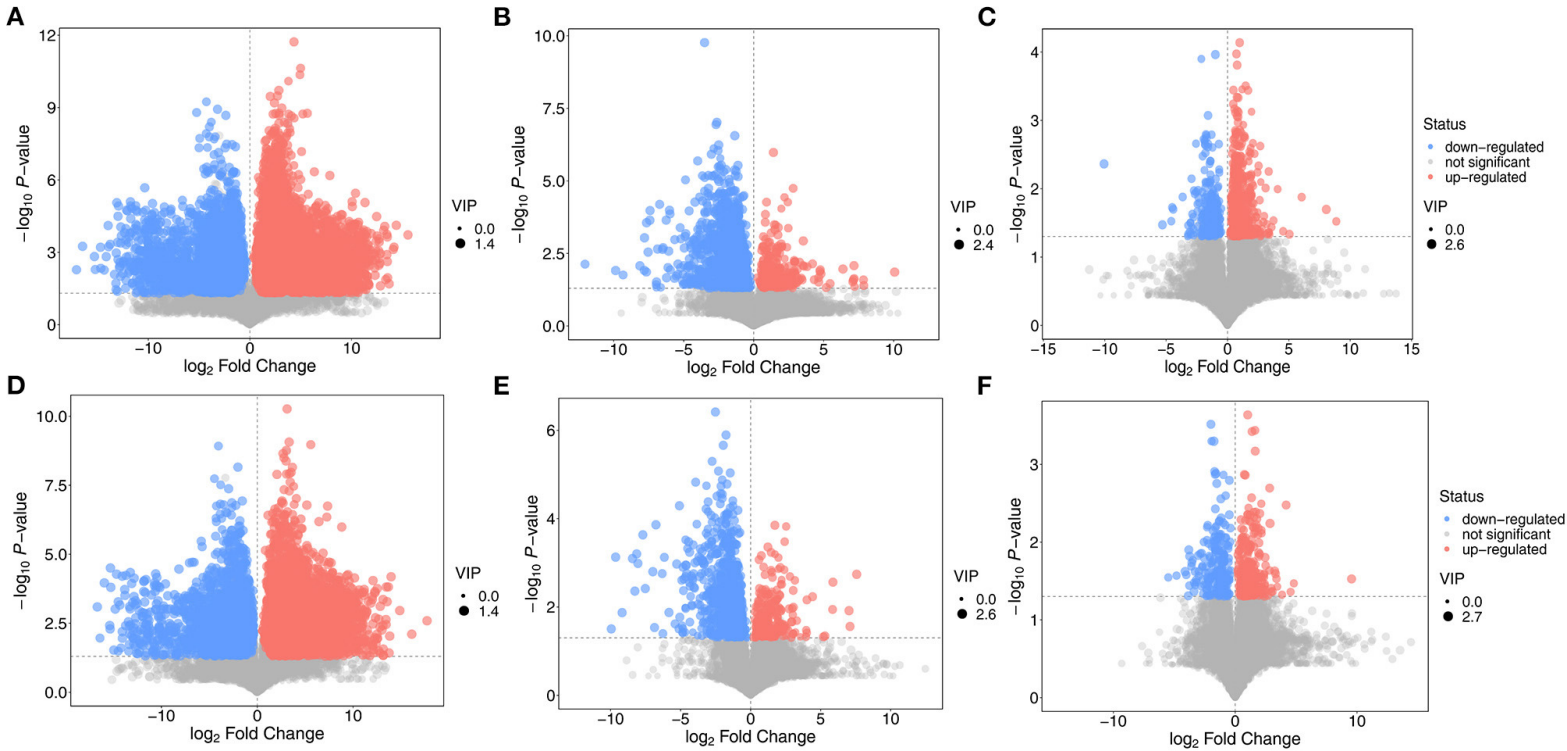
Volcano plot of differential expressed metabolite in feces of mice. **(A,D)**: NT-free group compared with NT intervention group; **(B,E)**: basal diet group compared with NT intervention group; **(C,F)**: basal diet group compared with model control group.

The criterion for screening differentially expressed metabolites was that the *P*-value obtained from Student's *t*-test should be <0.05. The variable importance in the projection of the first principal component of the OPLS-DA model was >1.0. A volcano map of the differentially expressed metabolites is shown in Figure 9. Compared with the NT intervention group, there were many upregulated and downregulated substances in the NT-free group, indicating that metabolites in the groups with and without NTs were greatly changed. There were significantly more downregulated substances in the basal diet group than in the NT intervention group, suggesting that some metabolites were significantly reduced after supplementation with NTs. The number of up-regulated substances in basal diet group was higher than that in the model control group, indicating that the number of metabolites in the SAMR1 group was larger than that in the SAMP8 group. The differentially expressed metabolites in each group are shown in Table 1.

**Table 1:** Different expressed metabolites in feces of each group in positive ion mode.

**Group**	**MS2 name**	**MS2 score**	**VIP**	***P*-value**	**Fold change**	**Changing trend**
NT-free group compared with NT intervention group	Choline	1.000	1.219	0.000	0.389	↓^**^
	Ornithine	0.999	1.213	0.019	10.019	↑^*^
	D-Proline	0.998	1.161	0.016	4.114	↑^*^
	L-Lysine	0.998	1.111	0.020	2.898	↑^*^
	Deoxyadenosine	0.993	1.038	0.000	0.156	↓^**^
	L-Methionine	0.989	1.036	0.002	1.804	↑^**^
	Urocanic acid	0.987	1.130	0.019	0.248	↓^*^
	Riboflavin	0.983	1.070	0.004	0.348	↓^**^
	3-Methylhistidine	0.964	1.022	0.034	0.224	↓^*^
	Anserine	0.963	1.237	0.003	0.138	↓^**^
	Pyridoxine	0.960	1.364	0.000	0.022	↓^**^
	Hexanoylglycine	0.958	1.192	0.012	0.137	↓^*^
	Pyridoxal	0.950	1.304	0.000	0.327	↓^**^
	L-Arginine	0.948	1.212	0.001	3.846	↑^**^
	5-Hydroxyindoleacetic acid	0.940	1.330	0.002	0.044	↓^**^
	Adenosine	0.939	1.095	0.000	0.055	↓^**^
	Trigonelline	0.937	1.307	0.000	0.274	↓^**^
	4-Pyridoxic acid	0.910	1.177	0.000	0.132	↓^**^
	Methyldopa	0.891	1.330	0.000	0.204	↓^**^
	5,6-Dihydroxyindole	0.818	1.286	0.000	0.071	↓^**^
	N-Acetylleucine	0.817	1.138	0.001	0.434	↓^**^
	Lutein	0.807	1.322	0.000	0.109	↓^**^
	Indole	0.803	1.352	0.000	5.709	↑^**^
	Methylimidazole acetaldehyde	0.749	1.210	0.000	3.342	↑^**^
	Imidazole-4-acetaldehyde	0.744	1.163	0.000	0.353	↓^**^
	Linoleic acid	0.638	1.248	0.000	4.456	↑^**^
	Formiminoglutamic acid	0.510	1.334	0.001	0.177	↓^**^
	13S-hydroxyoctadecadienoic acid	0.409	1.256	0.005	9.038	↑^**^
Basal diet group compared with NT intervention group	2-Fucosyllactose	0.995	1.637	0.035	0.485	↓^*^
	1-Methylhistamine	0.978	1.504	0.030	0.540	↓^*^
	L-Proline	0.977	1.818	0.009	0.622	↓^**^
	Pyridoxal	0.950	1.886	0.002	0.639	↓^**^
	Thymine	0.928	1.391	0.041	0.684	↓^*^
	Methyldopa	0.891	2.225	0.000	2.854	↑^**^
	6,7-Dihydro-5-methyl-5H-cyclopenta[b]pyrazine	0.870	1.364	0.050	0.576	↓^*^
	4-Hydroxy-2-butenoic acid gamma-lactone	0.836	1.241	0.011	0.380	↓^**^
	Pyridoxal 5'-phosphate	0.822	2.160	0.000	0.191	↓^**^
Basal diet group compared with model control group	Deoxyguanosine	0.997	1.542	0.044	1.801	↑^*^
	Kynurenic acid	0.984	1.142	0.050	1.678	↑^*^
	Riboflavin	0.983	1.824	0.026	0.537	↓^*^
	L-Cyclo(alanylglycyl)	0.952	1.946	0.039	1.805	↑^*^
	Diethanolamine	0.945	1.848	0.037	3.572	↑^*^
	Cholesterol	0.915	1.315	0.041	0.593	↓^*^
	L-Threo-3-Phenylserine	0.836	1.554	0.036	1.590	↑^*^
	4-Hydroxy-2-butenoic acid gamma-lactone	0.836	1.958	0.030	467.029	↑^*^
	4-Aminohippuric acid	0.822	1.758	0.027	1.738	↑^*^
	Serotonin	0.822	1.940	0.017	1.988	↑^*^
	Dihydrotestosterone	0.527	1.983	0.040	2.843	↑^*^

*In the table, MS2 name: the name of the substance obtained from the secondary mass spectrometry qualitative matching analysis; MS2 score: the score of the secondary matching*.

* *Significant difference*;

** *extremely significant difference*.

**Figure 10:**
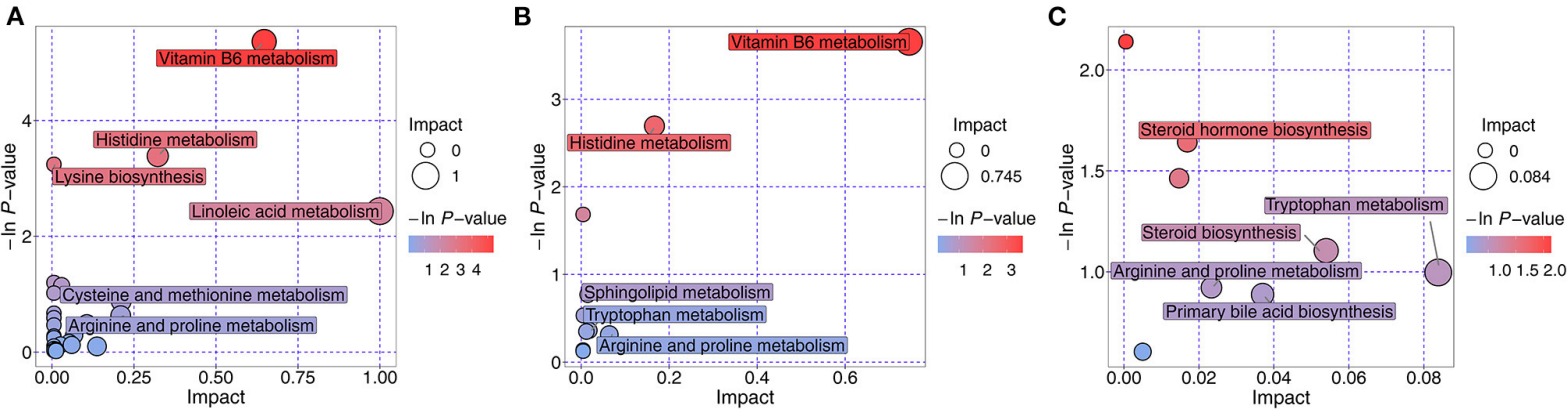
Metabolic pathway analysis of differentially expressed metabolites in the feces of mice. **(A)**: NT-free group compared with NT intervention group; **(B)**: basal diet group compared with NT intervention group; **(C)**: basal diet group compared with model control group.

The metabolic pathways of the differentially expressed metabolites were analyzed, and the results are shown in a bubble chart (Figure 10). The important paths were labeled through enrichment and topology analyses. Compared with the NT intervention group, 27 metabolic pathways were mainly affected by exogenous NTs in NT-free group. Vitamin B6 metabolism, histidine metabolism, and linoleic acid metabolism were significantly affected by exogenous NTs. Compared with the NT intervention group, 11 metabolic pathways were affected by exogenous NTs, particularly vitamin B6 and histidine metabolism. Compared with the model control group, accelerated aging affected nine metabolic pathways, including steroid hormone biosynthesis, steroid biosynthesis, and tryptophan metabolism (Supplementary Table 4).

### Correlation Analysis

**Figure 11:**
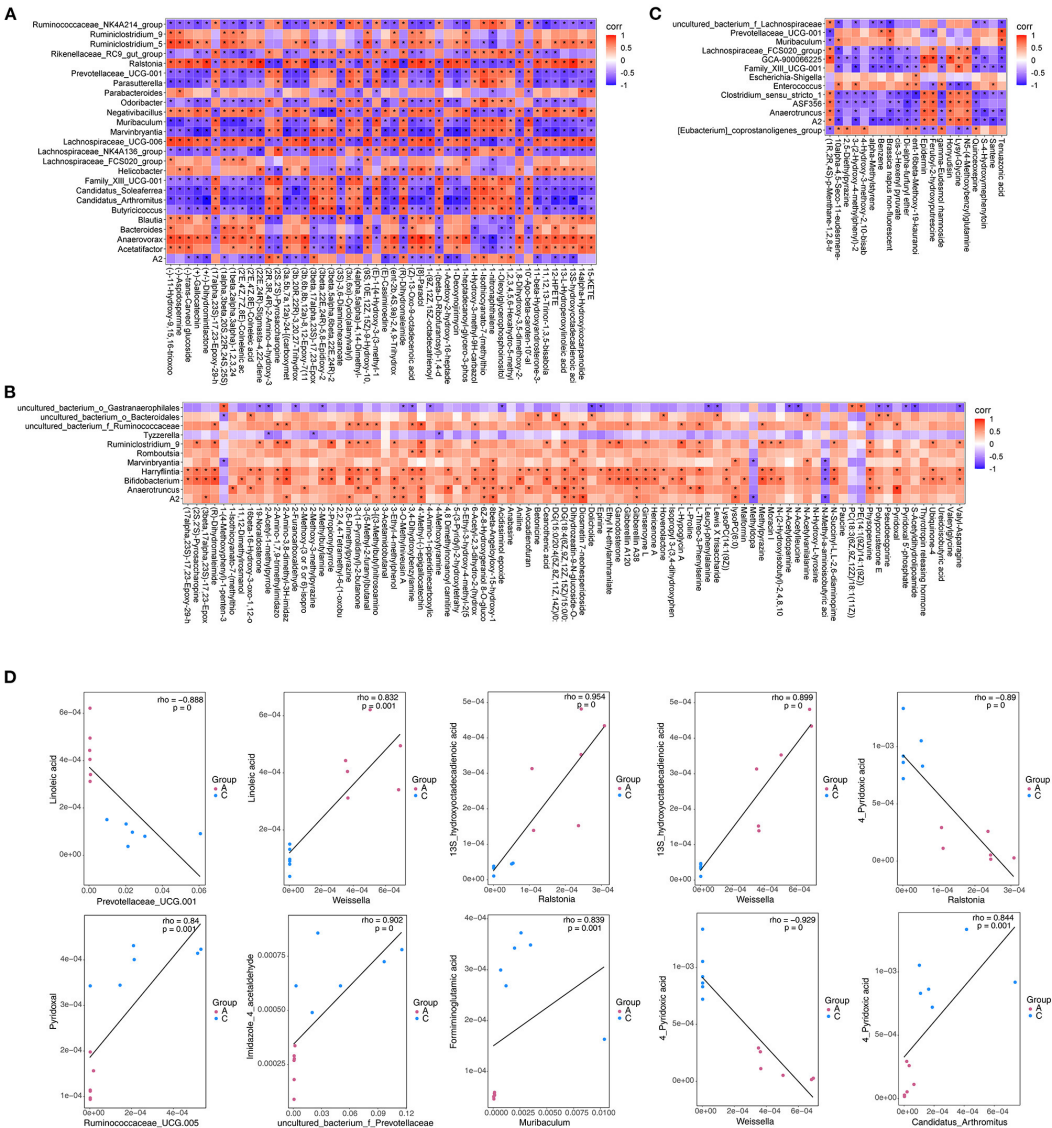
Thermogram of the correlation **(A–C)** and scatter plot **(D)** of differentially expressed microflora and differentially expressed metabolites in mouse feces. **(A)**: Partial screenshots of correlation analysis results between NT-free group and NT intervention group; **(B)**: correlation analysis results between basal diet group and NT intervention group; **(C)**: correlation analysis results between basal diet group and model control group.

To explore the functional correlation between differentially expressed bacteria and differentially expressed metabolites in fecal samples, correlation analysis was performed, and a thermogram was drawn to show the results (Figures 11A–C). Scatter plot analysis further verified the authenticity of the correlation coefficient analysis with microorganisms/metabolites to remove the strong correlation effect of false-positives (Figure 11D). There was an obvious interaction between differentially expressed bacterial groups and differentially expressed metabolites. For example, linoleic acid had a significant negative correlation with Prevotellaceae_UCG-001 (Corr = −0.89, *P* < 0.01) and significant positive correlation with Weissella (Corr = 0.83, *P* < 0.01); 13S-hydroxyoctadecadienoic acid was positively correlated with Ralstonia and Weissella (Corr = 0.95 and 0.90, *P* < 0.01). There was a significant positive correlation between pyridoxal and Ruminococcaceae_UCG-005 (Corr = 0.84, *P* < 0.01); 4-pyridoxic acid was negatively correlated with Anaerovorax and Ralstonia (Corr = −0.89, *P* < 0.01) but positively correlated with CandidatusArthromitus (Corr = 0.84, *P* < 0.01). Imidazole-4-acetaldehyde was significantly positively correlated with uncultured_bacterium_f_Prevotellaceae (Corr = 0.90, *P* < 0.01). Formiminoglutamic acid was also significantly positively correlated with Muribaculum (Corr = 0.84, *P* < 0.01). Therefore, addition of exogenous NTs to the diet of mice can significantly change the gut microbiota. The different flora were significantly correlated with metabolites, indicating that addition of exogenous NTs interfered with the abundance and altered the metabolic expression of the gut microbiota.

### Body Weight and Grading Score of Mice

**Figure 12:**
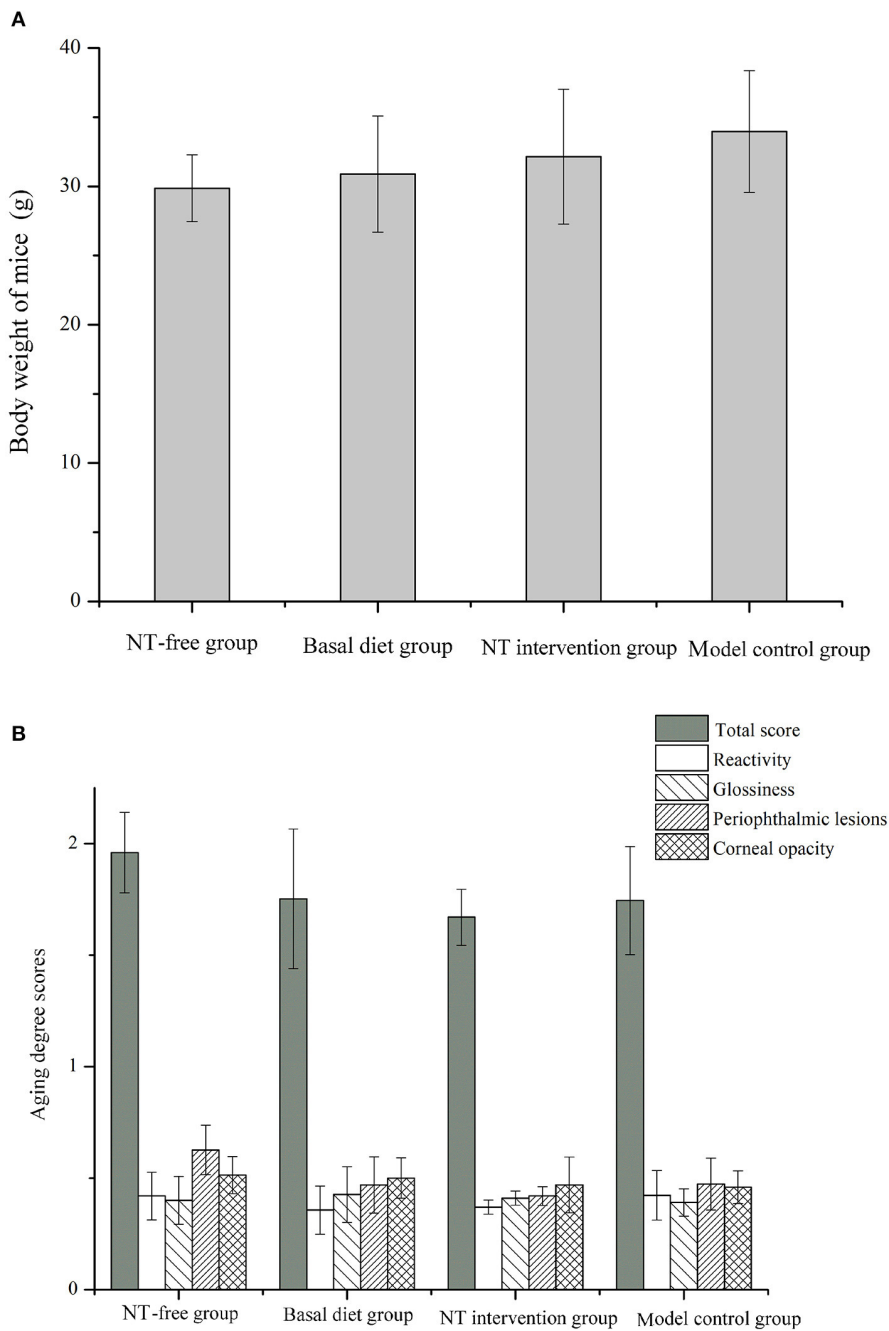
The body weight **(A)** and aging degree scores **(B)** of mice in four groups.

The body weight and grading score of mice were shown in Figures 12A,B. As can be seen from the figure, there was no significant difference in the weight of mice in the four groups, and there was no significance in mouse grading scores, such as reactivity, glossiness, periophthalmic lesions and corneal opacity. The weight of mice was the lowest in the NT-free group. In grading score of mice, total score, periophthalmic lesions and corneal opacity were the highest in the NT-free group, while the total score and periophthalmic lesions score were the lowest in the NT intervention group, indicating that the supplementation of NTs would bring some benefits to the mice, which might be related to the fact that NTs could improve the gut microbiota of mice.

### *In vitro* Experimental Results

**Figure 13:**
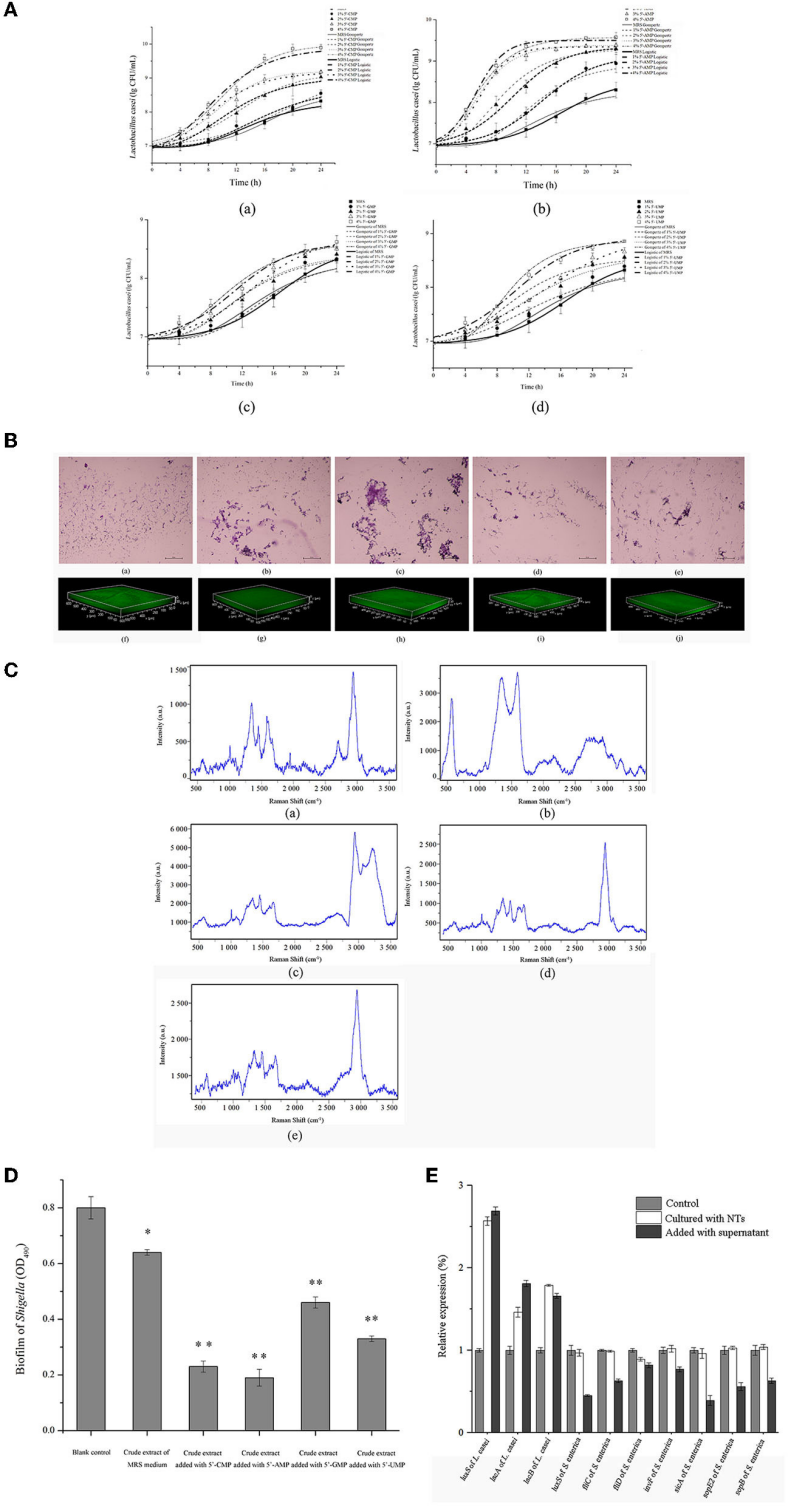
Effects of dietary NTs on the growth of *L. casei in vitro*. **(A)** The growth curve of *L. casei* was fitted by Modified Gompertz and Modified Logistic equation. **(a)** Addition of 5'-CMP; **(b)** Addition of 5'-AMP; **(c)** Addition of 5'-GMPNa_2_; **(d)** Addition of 5'-UMPNa_2_. **(B)** The effect of NTs on the biofilm of *L. casei* observed by optical microscope **(a–e)** and CLSM **(f–j)**. **(a, f)** Control; **(b, g)** Addition of 4.0% 5'-CMP; **(c, h)** Addition of 4.0% 5'-AMP; **(d, i)** Addition of 4.0% 5'-GMPNa_2_; **(e, j)** Addition of 4.0% 5'-UMPNa_2_. **(C)** Effect of NTs on the content and chemical composition of EPS produced by *L. casei*. **(a)** Control; **(b)** Addition of 5'-CMP; **(c)** Addition of 5'-AMP; **(d)** Addition of 5'-GMPNa_2_; **(e)** Addition of 5'-UMPNa_2_. **(D)** Effect of NTs on anti-biofilm activity of crude extracts of *L. casei*. **(E)** Results of RT-qPCR. ^*^p < 0.05, ^**^p < 0.001.

The effects of NTs on *L. casei* was studied by *in vitro* experiments. As shown in Figure 13A, *L. casei* grew faster in MRS medium supplemented with NTs than the blank control. The growth curve of bacteria was non-linear fitted by microbial growth kinetic model (Supplementary Table 5), and the maximum specific growth rate (μ_max_) of bacteria after adding NTs increased compared with the blank control, and the delay period (λ) was shortened. These results indicated that NTs could promote the growth of *L. casei*. In Figure 13B, after adding NTs to the culture medium of *L. casei*, the secretion of bacterial biofilm increased, and the bacteria became aggregated from dispersed state. The biofilm thickness of the control group was 30 μm. After adding 5'-CMP, 5'-AMP, 5'-GMPNa_2_ and 5'- UMPNa_2_, the biofilm increased to 55 μm, 70 μm, 35 μm and 40 μm. The results showed that NTs promoted the secretion of biofilm of *L. casei*. The effect of NTs on the content and chemical composition of EPS of *L. casei* was measured by Raman spectroscopy. It can be seen from Figure 13C that the Raman peak intensity of EPS increased after the addition of NTs. By comparing the characteristic peaks of Raman spectra, the secretion of corresponding substances in EPS increased after the addition of NTs. Therefore, NTs could increase the content of EPS secreted by *L. casei*. Figure 13D shows the effect of crude extract of *L. casei* on the biofilm of *Shigella*. It can be seen from the figure that after adding the crude extract of *L. casei*, the content of biofilm secreted by *Shigella* decreased. When NTs were added to *L. casei* culture medium, the inhibitory effect of the crude extract of *L. casei* on *Shigella* biofilm increased significantly. These results showed that the addition of NTs increased the ability of *L. casei* to antagonize the biofilm of pathogenic bacteria. NTs were also added to the co culture system of *L. casei* and another pathogenic bacteria (*Salmonella enterica*) and the changes of the expression levels of genes were tested by RT-qPCR. Figure 13E showed that NTs increased the relative expression of *luxS* gene and bacteriocin synthesis gene (*lacA*) of *L. casei*, but had no significant effect on the expression of *luxS* gene, flagella gene (*fliC, fliD*) and virulence factor gene (*invF, sicA, sopE2, sopB*) of *S. enterica*. NTs were added to the culture medium of *S. enterica*, and the supernatant was taken after culture. It was found that the supernatant could significantly increase the expression of *luxS* gene and bacteriocin synthesis gene of *L. casei*, while the expression of *luxS* gene, flagella gene and virulence factor of *S. enterica* decreased after adding *L. casei* supernatant. The results suggested that the NTs could enhance the ability of probiotics to antagonize intestinal pathogens.

## Discussion

The gut is the most important defense organ of the body and absorbs nutrients and regulates the stability and health of the internal environment. Aging is accompanied by changes in the gut microbiota. Studies have shown that gut microbiota diversity is reduced in the elderly. Beneficial bacteria are reduced or disappear, and bacteria producing hydrogen sulfide and indigo substrate are increased, leading to a rapid decay process and production of more harmful substances in the intestinal tract of the elderly. Absorption of these substances accelerates the aging process (33). It has been reported that an imbalance in intestinal microorganisms in aging mice may lead to intestinal leakage and release bacterial products that cause inflammation, thus damaging immune system function and shortening the lifespan. Similar results were observed in a study of humans. Elderly individuals with high levels of inflammatory factors are more likely to be weak and develop dementia and cardiovascular diseases (34). Increasing the number of beneficial bacteria and decreasing number of harmful bacteria may delaying aging in the body.

Research on 16S rDNA microbial diversity is mainly performed in conserved regions of the nucleic acid sequence encoding ribosomal RNA. Species annotation of the gut microbiota in mouse feces revealed that at the phylum level, the relative abundance of Bacteroides and Firmicutes in the NT-free group decreased significantly compared to that in the other three groups. Under normal conditions, Firmicutes and Bacteroides are the dominant flora in the intestinal tract of mice, and the most beneficial bacteria in the intestinal tract belong to these two phyla (35). Parthasarathy et al. (36) found that some genera of Firmicutes (e.g., *Faecalibacterium, Lactococcus*, and *Roseburia*) promote colonic peristalsis. Roseburia can ferment a variety of carbohydrates and increase the content of butyric acid in the intestine, which is useful for preventing and treating obesity-related diseases. Therefore, a lack of NTs in the diet may reduce the abundance of Bacteroides and Firmicutes in the intestinal tract of mice. In addition, compared with the SAMR1 group, the abundance of Firmicutes was lower in the gut microbiota of SAMP8 mice, indicating that aging also affects the gut microbiota of mice. Notably, the abundance of Proteobacteria was also highest in the NT-free group (group A). Proteobacteria is the largest phylum of bacteria and includes many pathogenic bacteria, such as *Escherichia coli, Salmonella, Vibrio cholerae, and Helicobacter pylori*. It is the main bacterium that easily causes animal diarrhea and is a marker of a gut microbiota imbalance. The abundance of Proteobacteria can be used to characterize the unstable intestinal microbial community and resulting metabolic disorders (37, 38). These results suggest that the relative abundance of intestinal pathogens in mice increased because of a deficiency in dietary NTs. In addition, the abundance of Cyanobacteria, Epsilonbacteraeota and Verrucomicrobia was high in NT-free group, and they were also aging related bacteria, indicating that NTs deficiency in the diet of mice would increase the abundance of aging related bacteria. The blind mole (*Spalax leucodon*) has become a unique model organism because of its longevity, hypoxia tolerance, hypercapnia tolerance, and cancer resistance. Sibai et al. found that Muribaculaceae, Lachnospiraceae, and Ruminococcaceae were the most significant taxa related to the metabolic activity of blind moles. Lachnospiraceae and Ruminococcaceae play important roles in butyrate production, whereas Muribaculaceae is involved in propionic acid production. Short-chain fatty acids such as butyric acid and propionic acid play an essential role in maintaining homeostasis and enabling disease recovery in the host. Therefore, the composition of the core microbiota in the blind mole may explain the health and longevity of this animal (39). *Candidatus saccharimonas* exerts anti-inflammatory effects by secreting metabolites and maintaining intestinal homeostasis, and showed the highest abundance in the NT intervention group, suggesting that supplementation of NTs to the diet of mice could increase the abundance of these bacteria. *Helicobacter* is a gram-negative genus in Campylobacter that mainly causes digestive tract diseases. The representative strain is *Helicobacter pylori*, which is closely related to gastric sinusitis, duodenal ulcers, and gastric ulcers and may also be related to gastric cancer. The relative abundance of *Helicobacter* was highest in the NT-free group and lowest in the NT intervention group. The results showed that addition of exogenous NTs to the diet of mice reduced the abundance of *Helicobacter*. *Lactobacillus* belongs to the phylum Firmicutes and has beneficial effects on host health. The abundance of *Lactobacillus* was lowest in the NT-free group and highest in the SAMR1 group, indicating that NTs play important roles in the colonization of beneficial bacteria such as *Lactobacillus*.

Significant differences between groups were analyzed by Kruskal Wallis rank sum test, which showed that the relative abundance of Verrucomicrobia, Ruminococcaceae, and Akkermansia was highest in the NT-free group. Verrucomicrobia is found mainly in aquatic and soil environments, or in human feces. Ruminococcaceae is a gram-positive anaerobic bacterium found in the human body that can secrete β-glucuronidase. β-Glucuronidase can produce active substances through a series of reactions that destroy the colonic mucosa and participate in tumor invasion and metastasis (40, 41). These results showed that bacteria with negative effects on the body were enriched in the NT-free group (group A), whereas the beneficial bacteria, such as *Lactobacillus*, had the lowest abundance in the NT-free group. There is a relationship between the change in *Lactobacillus* flora structure and improvement in cognitive impairment in the aging process. Studies have shown that *Lactobacillus* can produce γ-aminobutyric acid (GABA) by metabolizing glutamic acid. GABA is an important inhibitory neurotransmitter in the central nervous system and plays a role in protecting cognition. Changes in *Lactobacillus* bacteria in the gut microbiota are related to changes in GABA levels, which may regulate learning and cognitive function, to resist cognitive impairment during aging (42–45). Therefore, a lack of NTs in the diet may affect cognitive impairment during aging.

Metabonomics mainly involves the study of changes in small-molecular metabolites (molecular weight below 1000 Da) in biological samples, which are involved in complex biochemical processes. The relationship between the gut microbiota and host metabolism has been widely examined; however, most of these were independent studies. There have been few studies on the relationship between changes in microflora and host metabolites. Therefore, we use a non-target metabonomics analysis method to screen differentially expressed metabolites and metabolic pathways to clarify the effect of exogenous NTs on the gut microbiota and metabolites in mice. Feces are co-metabolites of the gut microbiota and host, which can reflect the status of the gut microbiota to some extent. We observed significant differences in fecal metabolites between the NT-free and NT addition groups and between SAMP8 and SAMR1 mice. Correlation analysis showed a significant correlation between differentially expressed metabolites and the gut microbiota. The gut microbiota produces monosaccharides, short-chain fatty acids, and other metabolites by fermenting food. These metabolites can be absorbed by the body as an energy source. Therefore, addition of exogenous NTs to the diet affects the gut microbiota, resulting in changes in metabolites of the gut microbiota. Changes in intestinal metabolites reacted with the body to improve the gut microbiota of SAMP8 mice.

Based on analysis of the metabolic pathways involved in the significant differentially expressed metabolites in mouse feces, the possible changes in metabolic pathways in mice induced by the supplementation of exogenous NTs in the diet may be as follows: (1) Compared with the NT intervention group, an absence of NTs from the diet mainly affected linoleic acid metabolism. The metabolic pathway map shows that lecithin increased under the catalysis of secretory phospholipase A2, linoleate content increased, and 13(S)-HPODE and 13(S)-HODE increased accordingly. A lack of NTs in the mouse diet promoted the metabolism of this pathway. (2) Compared with the NT intervention group, vitamin B6 metabolism was affected by the absence of NTs in the mouse diet in the NT-free group. Pyridoxine (vitamin B6) was produced by pyridoxal under the action of pyridoxamine 5′-phosphate oxidase, and then transformed with pyridoxamine via the action of various enzymes, or 4-pyridoxate was formed under the action of aldehyde oxidase, after which it is excreted. In the NT-free group, pyridoxine, pyridoxal, pyridoxamine, and 4-pyridoxate decreased significantly, indicating that exogenous NT deficiency inhibited the metabolism of this pathway and significantly affected the metabolism of vitamin B6. (3) Compared with the NT intervention group, histidine metabolism was affected by the absence of NTs from the diet. The metabolic pathway map showed that L-histidine produced urocanate under the action of histidine ammonia-lyase, and N-formimino-L-glutamate was produced under the action of urocanate hydratase and imidazolonepropionase. The decrease in urocanate and N-formimino-L-glutamate indicated that this pathway was inhibited. In another pathway, histamine is produced by L-histidine via the action of histidine decarboxylase; thereafter, imidazole acetaldehyde is produced by diamine oxidase, or N-methylhistamine is produced by histamine N-methyltransferase, and methylimidazole acetaldehyde is produced via the action of monoamine oxidase. Although the level of imidazole acetaldehyde decreased, methylimidazole acetaldehyde increased significantly, possibly because the other two pathways were inhibited. (4) Compared with the model control group, tryptophan metabolism, steroid biosynthesis, and steroid hormone biosynthesis were mainly affected in SAMP8 mice. In tryptophan metabolism, serotonin increased significantly; in steroid biosynthesis metabolism, cholesterol decreased significantly; in steroid hormone biosynthesis metabolism, dihydrotestosterone increased significantly. Cholesterol is an indispensable nutrient for the human body and material basis of human cell membranes, sex hormones, and cortisol. It also plays an important role in white blood cell activity. Studies have shown that low cholesterol levels increase the risk of cerebral hemorrhage stroke and depression in the elderly. Low cholesterol levels can accelerate serotonin reuptake in the brain. The elderly are very sensitive to serotonin uptake in the brain, and serotonin has a significant inhibitory effect on central nervous system function, causing the elderly to be very vulnerable to depression (46). This may explain the significant increase in serotonin levels. Moreover, the significant increase in dihydrotestosterone may be related to the synthesis or release of dihydrotestosterone into the blood in the surrounding tissues.

Aging will cause imbalance of gut microbiota, resulting in the reduction of bacteria beneficial to the body. Therefore, in order to explore whether NTs had promoting effect on the growth of probiotics *in vitro, L. casei* was taken as the research object and NTs were used as the intervention. As one of the probiotics, *L. casei* is used as the starter of milk, yogurt and other dairy products. It is widely used in the development of functional foods, especially dairy products. *L. casei* can produce beneficial metabolites such as organic acids and antimicrobial peptides. It has important biological effects such as antibacterial, regulating gut microbiota balance, promoting digestion and absorption, regulating body immunity, anti-aging and anti-oxidation. In recent years, *L. casei* from different sources has been isolated and identified, and its prebiotic function has been studied and applied. However, when *L. casei* are processed, sold and passed through the digestive tract, they are often affected by adverse conditions, resulting in the easy inactivation of bacteria. Results showed that NTs significantly promoted the bacterial growth, secretion of biofilm and EPS, and promoted the ability of the crude extract of *L. casei* to resist the biofilm of *Shigella*. Therefore, exploring the promoting effect of NTs on the growth of *L. casei* can improve the survival rate of *L. casei* and has great market potential.

## Conclusion

In conclusion, analysis of 16S rDNA revealed that a lack of NTs in the diet reduced the diversity of the gut microbiota in SAMP8 mice, whereas addition of exogenous NTs promoted recovery of the microbial diversity in mice. In addition, bacteria with negative effects such as *Helicobacter* were enriched in the NT-free group, whereas those with beneficial effects such as *Lactobacillus* were lowest in the NT-free group. Metabonomics analysis showed that dietary NTs significantly affected the fecal metabolites in mice, and addition of NTs returned the metabolite levels to normal. Changes in the gut microbiota induced by exogenous NTs were significantly correlated with gut microbiota metabolites. In addition, NTs could promote the growth, secretion of biofilm and EPS of *L. casei*.

## Data Availability Statement

The datasets presented in this study can be found in online repositories. The names of the repository/repositories and accession number(s) can be found below: National Center for Biotechnology (NCBI) BioProject, https://www.ncbi.nlm.nih.gov/bioproject/, PRJNA790683, and European Molecular Biology Laboratory's European Bioinformatics Institute (EMBL-EBI) MetaboLights, https://www.ebi.ac.uk/metabolights/, MTBLS4012. The metabonomic data may not be readily available as it is in curation process, further inquiries can be directed to Ting Ding, dingting0532@163.com.

## Ethics Statement

The animal study was reviewed and approved by Peking University Biomedical Ethics Committee.

## Author Contributions

MX and YL conceived and designed the experiments. TD performed the experiment, analyzed the data, and drafted the manuscript. All authors contributed to the article and approved the submitted version.

## Conflict of Interest

In this study, Zhen-ao company provided a lot of technical information about NTs, and Zhen-ao company sells NTs as dietary supplement. We promise to avoid conflicts of interest with Zhen-ao company, and the paper has not been affected in the process of writing. We have fully disclosed these interests to the editor and have developed an approved plan to manage any potential conflicts that may arise from such an arrangement.

## Publisher's Note

All claims expressed in this article are solely those of the authors and do not necessarily represent those of their affiliated organizations, or those of the publisher, the editors and the reviewers. Any product that may be evaluated in this article, or claim that may be made by its manufacturer, is not guaranteed or endorsed by the publisher.

## Supplementary Material

Supplementary Figure 1 TIC diagram of mouse feces by UHPLC-QE-MS. **(a-d)**: positive ion mode; **(e-h)**: negative ion mode.

Supplementary Table 1 AIN-93M diet formulated for maintenance of mice. Mineral blend pack and vitamin blend pack were in the diet.

Supplementary Table 2 Basal diet formulated for maintenance of mice. The minerals and vitamins of basal diet are basically consistent with those of AIN-93M diet.

Supplementary Table 3 The NTs levels in NTs-free, basal and NTs-supplied diets.

Supplementary Table 4 Metabolic pathway analysis of exogenous SAMR1 mice. Metabolic pathway analysis of exogenous nucleotides on SARMP8 and SAMR1 mice.

Supplementary Table 5 Nonlinear parameters of Lactobacillus casei fitted by Modified Gompertz and Modified Logistic equation.

Supplementary Table 6 The Primer sequences of *L. casei* and *S. enterica*.
