# Supplementary figures and images for: An Overlooked Prebiotic: Beneficial Effect of Dietary Nucleotide Supplementation on Gut Microbiota and Metabolites in Senescence-Accelerated Mouse Prone-8 Mice
Source: Front Nutr. 2022 Mar 24;9:820799. doi: 10.3389/fnut.2022.820799 (PMC8988891; doi:10.3389/fnut.2022.820799)

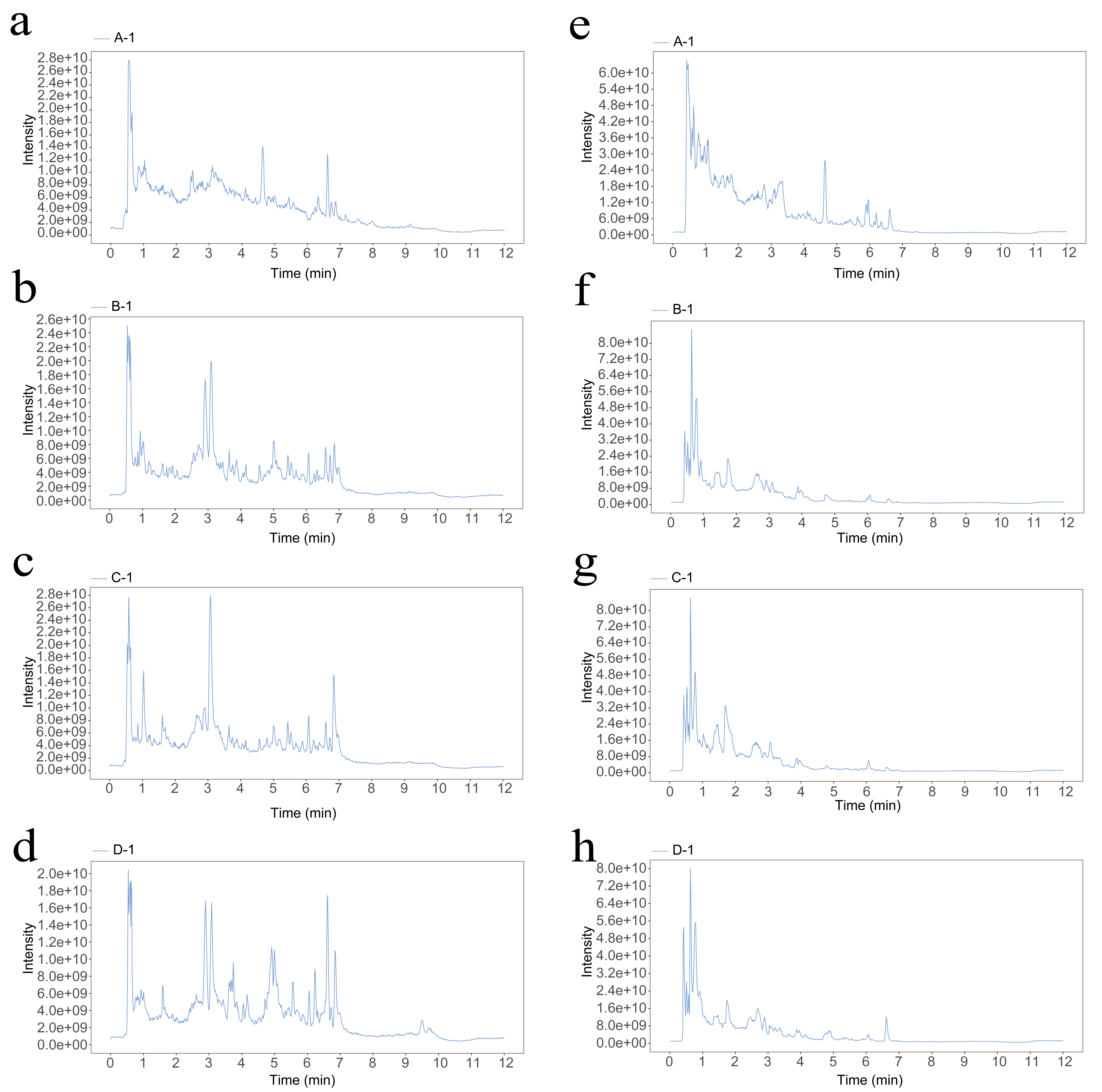

Supplement: Supplementary Figure 1 — TIC diagram of mouse feces by UHPLC-QE-MS. (a-d): positive ion mode; (e-h): negative ion mode. [file Image_1.TIF]
