# Supplementary material for: An Overlooked Prebiotic: Beneficial Effect of Dietary Nucleotide Supplementation on Gut Microbiota and Metabolites in Senescence-Accelerated Mouse Prone-8 Mice
Source: Front Nutr. 2022 Mar 24;9:820799. doi: 10.3389/fnut.2022.820799 (PMC8988891; doi:10.3389/fnut.2022.820799)
Supplement: Supplementary Table 1 — AIN-93M diet formulated for maintenance of mice. Mineral blend pack and vitamin blend pack were in the diet. [file Table_1.DOCX]

**Table S1** AIN-93M diet formulated for maintenance of mice

| Ingredient | g/kg diet |
| --- | --- |
| Cornstarch | 46.5692 |
| Casein (≥85% protein) | 14 |
| Maltodextrin | 15.5 |
| Sucrose | 10 |
| Soybean oil (no additives) | 4 |
| Fiber | 5 |
| Mineral mix (AIN-93M-MX) | 3.5 |
| Vitamin mix (AIN-93M-VX) | 1 |
| Choline bitartrate (41.1% choline) | 0.25 |
| BHT | 0.0008 |

Mineral blend pack and vitamin blend pack were in the diet.
