# Supplementary material for: An Overlooked Prebiotic: Beneficial Effect of Dietary Nucleotide Supplementation on Gut Microbiota and Metabolites in Senescence-Accelerated Mouse Prone-8 Mice
Source: Front Nutr. 2022 Mar 24;9:820799. doi: 10.3389/fnut.2022.820799 (PMC8988891; doi:10.3389/fnut.2022.820799)
Supplement: Supplementary Table 2 — Basal diet formulated for maintenance of mice. The minerals and vitamins of basal diet are basically consistent with those of AIN-93M diet. [file Table_2.DOCX]

**Table S2** Basal diet formulated for maintenance of mice

| Ingredient | g/kg diet |
| --- | --- |
| Methionine + Cystine, (g) | 5.80 |
| Lysine, (g) | 8.90 |
| Tryptophan, (g) | 2.10 |
| Arginine, (g) | 9.90 |
| Leucine, (g) | 14.80 |
| Isoleucine, (g) | 7.40 |
| Threonine, (g) | 6.60 |
| Valine, (g) | 8.90 |
| Histidine, (g) | 4.90 |
| Phenylalanine + Tyrosine, (g) | 14.60 |
| Vitamin A, IU | 7800.00 |
| Vitamin D, IU | 1200.00 |
| Vitamin E, mg | 67.00 |
| Vitamin K, mg | 5.00 |
| Vitamin B1, mg | 10.00 |
| Vitamin B2, mg | 15.00 |
| Vitamin B6, mg | 10.00 |
| Vitamin B12, mg | 0.02 |
| Niacin, mg | 55.00 |
| Pantothenic acid, mg | 22.00 |
| Biotin, mg | 0.20 |
| Choline, mg | 1250.00 |
| Folic acid, mg | 6.60 |
| Na, (g) | 3.10 |
| Mg, (g) | 2.90 |
| K, (g) | 7.40 |
| Cu, mg | 11.40 |
| Fe, mg | 113.70 |
| Mn, mg | 80.00 |
| Zn, mg | 31.60 |
| Se, mg | 0.20 |
| I, mg | 0.70 |

The minerals and vitamins of basal diet are basically consistent with those of AIN-93M diet.
