# Supplementary material for: An Overlooked Prebiotic: Beneficial Effect of Dietary Nucleotide Supplementation on Gut Microbiota and Metabolites in Senescence-Accelerated Mouse Prone-8 Mice
Source: Front Nutr. 2022 Mar 24;9:820799. doi: 10.3389/fnut.2022.820799 (PMC8988891; doi:10.3389/fnut.2022.820799)
Supplement: Supplementary Table 3 — The NTs levels in NTs-free, basal and NTs-supplied diets. [file Table_3.DOCX]

**Table S3** The NTs levels in NTs-free, basal and NTs-supplied diets

| Group | NT-free group | basal diet group | NT intervention group | Model control group |
| --- | --- | --- | --- | --- |
| diet | purified diet | basal diet | basal diet | basal diet |
| C | 0 | 95.4 | 614.3 | 95.4 |
| A | 0 | 348 | 581.6 | 348 |
| U | 0 | 714.3 | 1084.9 | 714.3 |
| G | 0 | 328.5 | 712.2 | 328.5 |
| Total NTs content (mg/kg) | 0 | 1486.2 | 2993 | 1486.2 |
