# Supplementary material for: An Overlooked Prebiotic: Beneficial Effect of Dietary Nucleotide Supplementation on Gut Microbiota and Metabolites in Senescence-Accelerated Mouse Prone-8 Mice
Source: Front Nutr. 2022 Mar 24;9:820799. doi: 10.3389/fnut.2022.820799 (PMC8988891; doi:10.3389/fnut.2022.820799)
Supplement: Supplementary Table 4 — Metabolic pathway analysis of exogenous SAMR1 mice. Metabolic pathway analysis of exogenous nucleotides on SARMP8 and SAMR1 mice. [file Table_4.DOCX]

**Table S4** Metabolic pathway analysis of exogenous nucleotides on SARMP8 and SAMR1 mice

| Group | Pathway | Total | Hits | Raw p | -ln(p) | Holm adjust | FDR | Impact |
| --- | --- | --- | --- | --- | --- | --- | --- | --- |
| NT-free group compared with basal diet group | Linoleic acid metabolism | 6 | 2 | 0.08766 | 2.4343 | 1 | 1 | 1 |
|  | Vitamin B6 metabolism | 9 | 4 | 0.0046905 | 5.3622 | 0.38462 | 0.38462 | 0.64706 |
|  | Histidine metabolism | 15 | 4 | 0.03384 | 3.3861 | 1 | 1 | 0.32258 |
| Basal diet group compared with NT intervention group | Vitamin B6 metabolism | 9 | 2 | 0.025861 | 3.655 | 1 | 1 | 0.7451 |
|  | Histidine metabolism | 15 | 2 | 0.06762 | 2.6938 | 1 | 1 | 0.16667 |
| Basal diet group compared with model control group | Tryptophan metabolism | 40 | 1 | 0.36912 | 0.99664 | 1 | 1 | 0.08384 |
|  | Steroid biosynthesis | 35 | 1 | 0.33124 | 1.1049 | 1 | 1 | 0.05394 |
|  | Steroid hormone biosynthesis | 72 | 2 | 0.1935 | 1.6425 | 1 | 1 | 0.01689 |

In the table, Pathway: the name of metabolic pathway; Total: the number of metabolites in the pathway; Hits: the number of differentially expressed metabolites hitting the pathway; Raw p: P value of metabolic pathway enrichment analysis; - ln (P): P value is the negative logarithm with e as the base; Holm adjust: P value corrected by Holm-Bonferroni multiple hypothesis test; FDR: P value corrected by multiple hypotheses test with false discovery rate (FDR); Impact: impact value of topological analysis of metabolic pathway.
