# Supplementary material for: An Overlooked Prebiotic: Beneficial Effect of Dietary Nucleotide Supplementation on Gut Microbiota and Metabolites in Senescence-Accelerated Mouse Prone-8 Mice
Source: Front Nutr. 2022 Mar 24;9:820799. doi: 10.3389/fnut.2022.820799 (PMC8988891; doi:10.3389/fnut.2022.820799)
Supplement: Supplementary Table 5 — Nonlinear parameters of Lactobacillus casei fitted by Modified Gompertz and Modified Logistic equation. [file Table_5.DOCX]

**Table S5** Nonlinear parameters of *Lactobacillus casei* fitted by Modified Gompertz and Modified Logistic equation

| Culture medium | Modified Gompertz | | | Modified Logistic | | | |
| --- | --- | --- | --- | --- | --- | --- | --- |
|  | μ_max_ (h^-1^) | λ(h) | R^2^ | A | μ_max_ (h^-1^) | λ(h) | R^2^ |
| MRS | 0.09047 | 6.84726 | 0.98329 | 1.54757 | 0.10172 | 8.88033 | 0.99828 |
| 5’-CMP (1%) | 0.08917 | 6.74498 | 0.91978 | 2.19258 | 0.11961 | 7.77916 | 0.978 |
| 5’-CMP (2%) | 0.1409 | 3.72084 | 0.98927 | 2.16845 | 0.13547 | 4.37309 | 0.99166 |
| 5’-CMP (3%) | 0.19268 | 2.71731 | 0.99393 | 2.2363 | 0.17275 | 2.8792 | 0.99356 |
| 5’-CMP (4%) | 0.21553 | 2.45227 | 0.98142 | 3.00934 | 0.20956 | 2.85966 | 0.97685 |
| 5’-AMP (1%) | 0.15697 | 6.53712 | 0.9807 | 2.18113 | 0.15019 | 6.83812 | 0.99461 |
| 5’-AMP (2%) | 0.209 | 2.90927 | 0.99192 | 2.39424 | 0.1772 | 3.46437 | 0.99473 |
| 5’-AMP (3%) | 0.29069 | 1.41773 | 0.99869 | 2.40113 | 0.29979 | 1.83778 | 0.99317 |
| 5’-AMP (4%) | 0.29439 | 0.91903 | 0.99875 | 2.55801 | 0.33416 | 1.46332 | 0.99417 |
| 5’-GMPNa_2_ (1%) | 0.0969 | 7.70922 | 0.93499 | 1.72785 | 0.10317 | 7.6688 | 0.98149 |
| 5’-GMPNa_2_ (2%) | 0.1 | 4.919 | 0.96762 | 1.62175 | 0.10444 | 5.18287 | 0.9937 |
| 5’-GMPNa_2_ (3%) | 0.10604 | 4.58761 | 0.9084 | 1.75846 | 0.11725 | 5.0273 | 0.9657 |
| 5’-GMPNa_2_ (4%) | 0.11815 | 3.29033 | 0.98757 | 1.66689 | 0.12136 | 3.47322 | 0.99087 |
| 5’-UMPNa_2_ (1%) | 0.09314 | 4.32895 | 0.97811 | 1.72929 | 0.10842 | 6.0875 | 0.99851 |
| 5’-UMPNa_2_ (2%) | 0.11562 | 3.4076 | 0.98403 | 1.77535 | 0.10892 | 4.27205 | 0.99749 |
| 5’-UMPNa_2_ (3%) | 0.12187 | 3.1489 | 0.97507 | 2.01416 | 0.11812 | 3.61412 | 0.99632 |
| 5’-UMPNa_2_ (4%) | 0.18806 | 3.03156 | 0.99708 | 1.97432 | 0.1271 | 2.47112 | 0.99934 |
