# Supplementary material for: An Overlooked Prebiotic: Beneficial Effect of Dietary Nucleotide Supplementation on Gut Microbiota and Metabolites in Senescence-Accelerated Mouse Prone-8 Mice
Source: Front Nutr. 2022 Mar 24;9:820799. doi: 10.3389/fnut.2022.820799 (PMC8988891; doi:10.3389/fnut.2022.820799)
Supplement: Supplementary Table 6 — The Primer sequences of L. casei and S. enterica. [file Table_6.DOCX]

**Table S6** The Primer sequences of *L. casei* and *S. enterica*

| Bacteria | gene | function | sequence | Primer TM value (℃) | Product length (bp) |
| --- | --- | --- | --- | --- | --- |
| *L. casei* | *16S* |  | F: GAGGCAGCAGTAGGGAATCT | 58.87 | 113 |
|  |  |  | R: GCCGACCATTCTTCTCCAAC | 58.91 |  |
|  | *lacA* | Synthetic bacteriocin | F: GGTGCAGACAAAGACGGATT | 58.48 | 102 |
|  |  |  | R: ATCTTCAGCAGGTTCAGGGG | 59.38 |  |
|  | *luxS* | Produce AI-2 | GCTTGCACACGATTGAACAC | 58.60 | 122 |
|  |  |  | GCTGTGCTCACCCCAAGTAA | 60.25 |  |
| *S. enterica* | *16S* |  | F: GCGCAACCCTTATCCTTTGT | 58.83 | 124 |
|  |  |  | R: TGTGTAGCCCTGGTCGTAAG | 59.11 |  |
|  | *luxS* | Produce AI-2 | F: ATCACCGTGTTTGATCTGCG | 58.92 | 244 |
|  |  |  | R: CTTTCAGCACATCCGCCATC | 59.62 |  |
|  | *fliC* | Flagellum gene | F: GCTGCTACAACCACCGAAAA | 59.05 | 137 |
|  |  |  | R: TTGTTTACGGTGTTGCCCAG | 58.97 |  |
|  | *fliD* | Flagellum gene | F: GCGAAGAAGCCAGCGATAAA | 58.99 | 135 |
|  |  |  | R: TGATGCCAATTTCCGCCATT | 58.81 |  |
|  | *invF* | Virulence factor | F: CGTTGTCGCACCAGTATCAG | 59.01 | 144 |
|  |  |  | R: TCAGGACTCAGCAAAACCCA | 59.16 |  |
|  | *sicA* | Virulence factor | F: TGGGATGCCGTTAGTGAAGG | 59.75 | 127 |
|  |  |  | R: ACGTCTCAGCTTCATCCAGT | 58.74 |  |
|  | *sopE2* | Virulence factor | F: ACCCAGCACTACAGAATCCA | 58.34 | 203 |
|  |  |  | R: CTACCCTCAGAAGCGTTCCC | 59.82 |  |
|  | *sopB* | Virulence factor | F: AACCACTCGCTGCATAACCT | 59.68 | 133 |
|  |  |  | R: GGTCCGCTTTAACTTTGGCT | 58.76 |  |
